# Detecting overlapping protein complexes based on a generative model with functional and topological properties

**DOI:** 10.1186/1471-2105-15-186

**Published:** 2014-06-13

**Authors:** Xiao-Fei Zhang, Dao-Qing Dai, Le Ou-Yang, Hong Yan

**Affiliations:** 1 School of Mathematics and Statistics, Central China Normal University, Luoyu Road, 430079 Wuhan, China; 2 Intelligent Data Center and Department of Mathematics, Sun Yat-Sen University, Xingang Road West, 510275 Guangzhou, China; 3 Department of Electronic Engineering, City University of Hong Kong, Tat Chee Avenue, Hong Kong, China

**Keywords:** Protein complex detection, Protein-protein interaction network, Functional profile, Generative model

## Abstract

**Background:**

Identification of protein complexes can help us get a better understanding of cellular mechanism. With the increasing availability of large-scale protein-protein interaction (PPI) data, numerous computational approaches have been proposed to detect complexes from the PPI networks. However, most of the current approaches do not consider overlaps among complexes or functional annotation information of individual proteins. Therefore, they might not be able to reflect the biological reality faithfully or make full use of the available domain-specific knowledge.

**Results:**

In this paper, we develop a Generative Model with Functional and Topological Properties (GMFTP) to describe the generative processes of the PPI network and the functional profile. The model provides a working mechanism for capturing the interaction structures and the functional patterns of proteins. By combining the functional and topological properties, we formulate the problem of identifying protein complexes as that of detecting a group of proteins which frequently interact with each other in the PPI network and have similar annotation patterns in the functional profile. Using the idea of link communities, our method naturally deals with overlaps among complexes. The benefits brought by the functional properties are demonstrated by real data analysis. The results evaluated using four criteria with respect to two gold standards show that GMFTP has a competitive performance over the state-of-the-art approaches. The effectiveness of detecting overlapping complexes is also demonstrated by analyzing the topological and functional features of multi- and mono-group proteins.

**Conclusions:**

Based on the results obtained in this study, GMFTP presents to be a powerful approach for the identification of overlapping protein complexes using both the PPI network and the functional profile. The software can be downloaded from http://mail.sysu.edu.cn/home/stsddq@mail.sysu.edu.cn/dai/others/GMFTP.zip.

## Background

Detecting protein complexes, which is crucial for elucidating the structural and functional architecture of cells, has attracted a lot of attention in recent years. Well-known experimental methods such as tandem affinity purification with mass spectrometry [1] and protein-fragment complementation assay [2], even though they are effective, have low efficiency, low coverage, and are biased [3].

Due to the development of high-throughput techniques, a large number of physical protein-protein interactions (PPI) have been generated and accumulated, which paves the way for establishing or reconstructing the PPI networks [4,5]. Two proteins interacting with each other in such network probably provide an evidence that they belong to a common protein complex. This intuition inspires us to split the whole network into groups, which have more links within each group and fewer links between different groups, to reveal its intrinsic structure and global organization in terms of protein complexes. Recently, numerous computational approaches relying on different strategies (e.g., graph clustering [6], community detection [7,8]) have been proposed to detect complexes from the PPI network [3,9-16]. However, those methods have their own shortcomings inevitably, since they only use the network topology.

**Figure 1:**
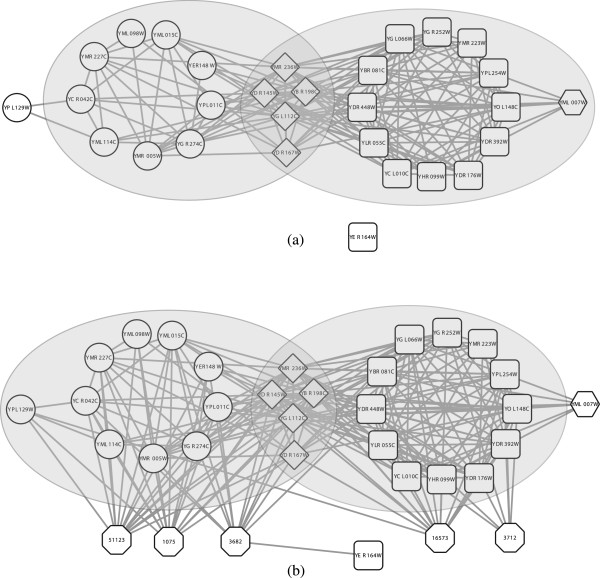
**An example which illustrates the biological motivation.** This is an interaction map of SAGA complex, transcription factor TFIID complex (CYC2008 benchmark) and the complexes detected by our model that match with them on Gavin network. Proteins are labeled according to the complex(es) to which they belong: rectangle represents SAGA complex; circle represents transcription factor TFIID complex; diamond represents proteins shared by the two complexes; octagon represents proteins with other functions; and hexagon represents GO terms. Shaded areas represent complexes detected by our model **(a)** using solely the PPI network and **(b)** using both the PPI network and the total GO annotation.

Proteins are often involved in more than one complex to serve different functions [17,18]; for example, there are five proteins (diamond nodes in Figure 1(a)) shared by the SAGA complex and the transcription factor TFIID complex according to the PPI data published in [4] and the CYC2008 benchmark [19]. However, traditional network clustering algorithms do not consider overlaps among complexes since each protein in the PPI network is assigned to only one complex. Therefore they are not able to fully reveal the biological reality. Furthermore, the PPI data produced by experimental bio-technology have a high level of noise and are incomplete [20,21]. The complexes predicted by a clustering algorithm based only on the PPI data may be limited in accuracy. For example, a complex detection approach may neglect protein YPL129W which is a member of the transcription factor TFIID complex due to the fewer interactions with the core members, and it may incorrectly cluster protein YML007W into the SAGA complex owing to the seven interactions (Figure 1(a)). Intuitively, proteins serving similar functions are more likely to belong to the same complex(es) than those serving different functions (Figure 1(b)). We wonder whether the functional annotations can work together with the PPI data to improve the quality of detected complexes; for example, to filter out functional heterogeneity protein YML007W and to retrieve functional homogeneity protein YPL129W.

In order to reduce the negative effect brought by the spurious interactions, several researchers have tried to incorporate functional information into complex detection process. These approaches can be mainly classified into two categories, preprocess-based [22-25] and postprocess-based [26,27]. The main idea of the former category is to design a functional semantic similarity measure to weight the strengths of protein-protein interactions, and then use a graph clustering algorithm to detect complexes from the weighted PPI network. They require the clustering algorithms to be able to handle weighted networks. However, there are only a few network clustering algorithms that can handle weights and overlaps simultaneously [17,28-32]. Furthermore, their performances depend on how the semantic measure is defined to assign the weights, which itself has many open problems [33]. The postprocess-based approaches use some metrics to quantify the functional homogeneity of complex candidates detected by graph clustering algorithms, and then discard candidates with low reliability. They do not make full use of the available functional annotations since such information are excluded during the complex candidate detection process. Recently, Zhang et al. map the topological and functional features into a unified distance measure by constructing an ontology augmented network, while they do not pay attention to the overlap problem [34].

As an alternative, we couple the functional profile with the network topology to detect overlapping protein complexes. To this end, we resort to probabilistic models which have been applied to analyze PPI networks [20,21,35-37]. Unlike previous models that account only for the generative process of the PPI network, we develop a new Generative Model with Functional and Topological Properties (GMFTP), which is dominated by two latent variables. One is introduced to represent the degree of proteins belonging to complex(es). By the idea of link communities [38,39], we generate a complex type-related interaction between two proteins if they tend to belong to the same complex(es). It gives rise to overlaps in a natural way that a protein belongs to multiple complexes if it has more than one type of interactions. The other one is used to represent the preferences of functions with which proteins in a complex associate. We generate an association between a protein and a function using these two model parameters. According to the introduced model, a complex is assumed to be a group of proteins which frequently interact with each other and have similar functional patterns. For a given PPI network and functional profile, we then transform the complex detection problem into a parameter estimation problem. We investigate the performance of our model using six yeast PPI networks and four categories of functional profiles. Experiment results show that the functional properties are able to improve the performance. Comparative experiments further demonstrate that our model not only has a better performance than the state-of-the-art approaches but also is capable of identifying proteins in multiple complexes.

## Methods

### A generative model with functional and topological properties

Before introducing our model, we introduce some notations first. We consider the functional and topological properties of *N* proteins. Each protein *i* has an annotation profile of fixed length *C*, *F*_*i*_ = [*F*_*i*1_,…,*F*_*i* *C*_]^*T*^ ∈ {0,1}^*C*^, where *F*_*ic*_ = 1 if protein *i* is associated with function *c*, *F*_*ic*_ = 0 otherwise, and *C* is the total number of functions considered. For convenience, we denote *F* = [*F*_1_,…,*F*_*N*_]^*T*^ = [*F*_*ic*_] ∈ {0,1}^*N*×*C*^ as the functional profiles for all proteins. The PPI network is represented as an adjacency matrix *A* = [*A*_*ij*_] ∈ {0,1}^*N*×*N*^, where *A*_*ij*_ = 1 if proteins *i* and *j* are connected, *A*_*ij*_ = 0 otherwise. We assume that there are *K* complexes. In the typical model-based clustering setting, the value of *K* is initially unknown and needs to be predetermined. Here we assume that the value is given first and address how to set it at the end of this section.

GMFTP generates both the annotation *F*_*ic*_ and the interaction *A*_*ij*_ as follows. In a similar manner to that of [37,39], a non-negative parameter *θ*_*ik*_ is introduced to represent the affinity of protein *i* belonging to complex *k*. A higher affinity score *θ*_*ik*_ means that protein *i* is more likely to belong to complex *k*, and vice versa. Note that a protein may obtain high affinity scores on multiple complexes, thus our model supports overlaps. Since proteins within the same complex(es) are always associated with same functions [40], for a given complex *k*, we introduce a non-negative parameter *ψ*_*kc*_ to represent the propensity that proteins in complex *k* are associated with function *c*. A higher score *ψ*_*kc*_ means that proteins in complex *k* are more likely to be associated with function *c*, and vice versa. In effect, *ψ*_*kc*_ represents the preferences of functions with which proteins in complex *k* are associated. We denote Θ = [*θ*_*ik*_] as the protein-complex affinity matrix and Ψ = [*ψ*_*kc*_] as the complex-function preference matrix.

By the definitions of *θ*_*ik*_ and *ψ*_*kc*_, if protein *i* obtains higher affinity score *θ*_*ik*_ and complex *k* obtains higher preference score *ψ*_*kc*_, protein *i* is more likely to be associated with function *c*, and vice versa. Then *θ*_*ik*_*ψ*_*kc*_ can be assumed as the likelihood that protein *i* is associated with function *c* in terms of complex *k*. Taking into account all the *K* complexes, we can assume $\sum_{k=1}^{K}\theta_{\text{ik}}\psi_{\text{kc}}$ to be the total likelihood that protein *i* is associated with function *c*. Then the association *F*_*ic*_ between protein *i* and function *c* is independently generated by a Bernoulli distribution with success rate $\sigma \left(\sum_{k=1}^{K}\theta_{\text{ik}}\psi_{\text{kc}}\right)$, where *σ*(*x*) = 1 - *exp*(-*x*) is a function which maps the input argument from [0,+*∞*) to [0,1), ensuring that the result is a valid probability.

A protein complex in the PPI network is usually assumed to be a cohesively connected subnetwork which has many interactions within itself [41], hence two proteins which belong to the same complex(es) are likely to interact with each other. If two proteins *i* and *j* obtain high affinity scores *θ*_*ik*_ and *θ*_*jk*_, they would be connected in complex *k*. We therefore assume that *θ*_*ik*_*θ*_*jk*_ is the likelihood that proteins *i* and *j* are connected in terms of complex *k*, and that $\sum_{k=1}^{K}\theta_{\text{ik}}\theta_{\text{jk}}$ is the total likelihood that they interact in terms of all the *K* complexes. Then the interaction *A*_*ij*_ between them is independently generated by a Bernoulli distribution with success probability $\sigma \left(\sum_{k=1}^{K}\theta_{\text{ik}}\theta_{\text{jk}}\right)$. Here we use function *σ*(*x*) to map the likelihood to the probability.

It is well known that a protein usually belongs to one or several complexes; and a protein complex tends to be responsible for (or be significantly enriched with) a given set of biological functions. This means Θ and Ψ are sparse essentially. To model the sparsity property, we place an independent exponential distribution prior over each element *θ*_*ik*_ and *ψ*_*kc*_ with rate parameter *λ*, which is similar to the sparsity promoting prior in non-negative sparse coding [42,43]. The sparse restriction may lead all elements in some columns of Θ and rows of Ψ to 0 simultaneously, and hence the corresponding irrelevant complexes will disappear automatically.

**Figure 2:**
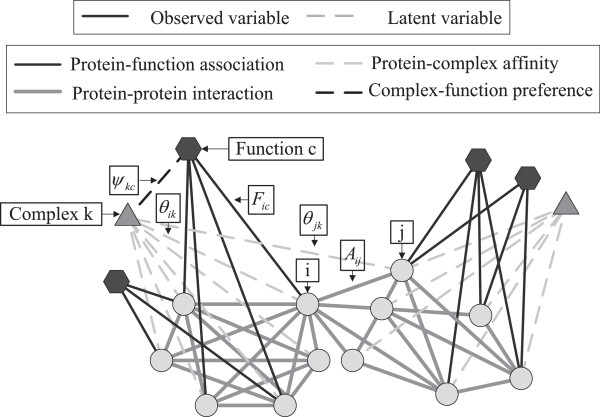
**A graphical representation of the connection between the variables we use and the biology terms.** Circle nodes represent proteins; triangle nodes represent complexes; hexagon nodes represent functions. We first introduce a model that generates protein-protein interaction *A*_*ij*_ and protein-function association *F*_*ic*_ based on model parameters *θ*_*ik*_ and *ψ*_*kc*_. For an observed PPI network and functional profile, we estimate the values of *θ*_*ik*_ and *ψ*_*kc*_. Finally, we predict protein complexes using the estimator of *θ*_*ik*_.

For a better understanding of our model, we illustrate the connection between the variables we use and the biology terms in Figure 2. Given hyperparameter *λ*, *N* proteins and *C* functional terms, the generative process of the functional profile and the PPI network with *K* complexes can be summarized as follows: 

• For each protein *i* and complex *k*, draw protein-complex affinity score *θ*_*ik*_ ∼ Exp(*λ*) with probability:

(1) $$P(\theta_{\text{ik}}|\lambda )=\lambda \exp\left(-\lambda \theta_{\text{ik}}\right),\;\theta_{\text{ik}}\geq 0.$$

• For each complex *k* and function *c*, draw complex-function preference score *ψ*_*kc*_ ∼ Exp(*λ*) with probability:

(2) $$P(\psi_{\text{kc}}|\lambda )=\lambda \exp\left(-\lambda \psi_{\text{kc}}\right),\;\psi_{\text{kc}}\geq 0.$$

• For each protein *i* and function *c*, sample their association value $F_{\text{ic}}∼\text{Bernoulli}\left(\sigma \left(\sum_{k=1}^{K}\theta_{\text{ik}}\psi_{\text{kc}}\right)\right)$ with probability:

(3) $$\begin{array}{l} P(F_{\text{ic}}|\Theta ,\Psi )={\left(\sigma \left(\overset{K}{\underset{k=1}{\sum }}\theta_{\text{ik}}\psi_{\text{kc}}\right)\right)}^{F_{\text{ic}}}{\left(1-\sigma \left(\overset{K}{\underset{k=1}{\sum }}\theta_{\text{ik}}\psi_{\text{kc}}\right)\right)}^{1-F_{\text{ic}}}. \end{array}$$

• For each pair of proteins *i* and *j* (*i* < *j*), sample their interaction value $A_{\text{ij}}∼\text{Bernoulli}\left(\sigma \left(\sum_{k=1}^{K}\theta_{\text{ik}}\theta_{\text{jk}}\right)\right)$ with probability:

(4) $$\begin{array}{l} P(A_{\text{ij}}|\Theta )={\left(\sigma \left(\overset{K}{\underset{k=1}{\sum }}\theta_{\text{ik}}\theta_{\text{jk}}\right)\right)}^{A_{\text{ij}}}{\left(1-\sigma \left(\overset{K}{\underset{k=1}{\sum }}\theta_{\text{ik}}\theta_{\text{jk}}\right)\right)}^{1-A_{\text{ij}}}. \end{array}$$

### Model formulation and parameter estimation

#### Model formulation

In previous section, we have introduced a generative process of the functional profile and the PPI network. Each run of this process generates a sample of the protein-complex affinity parameter Θ, complex-function preference parameter Ψ, functional profile *F* and PPI network *A*. Given the hyperparameter *λ*, we can decompose the joint probability distribution over *F*, *A*, Θ, Ψ using the dependent relationships stated in the previous definition and encoded in Figure S1 (in Additional file 1) as follows:

(5) P(F,A,Θ,Ψ|λ)=P(F|Θ,Ψ)P(A|Θ)P(Θ|λ)P(Ψ|λ),

where

(6) $$P(F|\Theta ,\Psi )=\overset{N}{\underset{i=1}{\prod }}{\left(\overset{C}{\underset{c=1}{\prod }}P(F_{\text{ic}}|\Theta ,\Psi )\right)}^{S_{i}},$$

(7) $$P(A|\Theta )=\underset{1\leq i<j\leq N}{\prod }P(A_{\text{ij}}|\Theta ),$$

(8) $$P(\Theta |\lambda )=\overset{N}{\underset{i=1}{\prod }}\overset{K}{\underset{k=1}{\prod }}P(\theta_{\text{ik}}|\lambda ),$$

(9) $$P(\Psi |\lambda )=\overset{K}{\underset{k=1}{\prod }}\overset{C}{\underset{c=1}{\prod }}P\left(\psi_{\text{kc}}|\lambda \right),$$

and *P*(*θ*_*ik*_|*λ*), *P*(*ψ*_*kc*_|*λ*), *P*(*F*_*ic*_|Θ,Ψ), *P*(*A*_*ij*_|Θ) are defined in Equations (1)-(4), respectively. Considering the case that the functional profiles of some proteins are not available, we introduce *S*_*i*_ to represent whether functional profile of protein *i* is generated, where *S*_*i*_ = 1 means the functional profile is generated, and *S*_*i*_ = 0 otherwise.

When the functional profile *F* and PPI network *A* are observed, we aim to find model parameters Θ and Ψ so that they maximize the likelihood *P*(*F*,*A*,Θ,Ψ|*λ*). By substituting Equations (1)-(4) into Equation (5), taking the negative logarithm and dropping constants, we formulate the objective function of GMFTP as follows:

(10) $$\begin{array}{l} \left\{\begin{array}{ll} \min_{\Theta ,\Psi } & -\sum_{i=1}^{N}\sum_{c=1}^{C}S_{i}F_{\text{ic}}\log\left(1-\exp\left(-\sum_{k=1}^{K}\theta_{\text{ik}}\psi_{\text{kc}}\right)\right)\\ & +\sum_{i=1}^{N}\sum_{c=1}^{C}S_{i}\left(1-F_{\text{ic}}\right)\left(\sum_{k=1}^{K}\theta_{\text{ik}}\psi_{\text{kc}}\right)\\ & -\frac{1}{2}\sum_{i,j=1}^{N}A_{\text{ij}}\log\left(1-\exp\left(-\sum_{k=1}^{K}\theta_{\text{ik}}\theta_{\text{jk}}\right)\right)\\ & +\frac{1}{2}\sum_{i,j=1}^{N}\left(1-A_{\text{ij}}\right)\left(\sum_{k=1}^{K}\theta_{\text{ik}}\theta_{\text{jk}}\right)\\ & +\sum_{i=1}^{N}\sum_{k=1}^{K}\lambda \theta_{\text{ik}}+\sum_{k=1}^{K}\sum_{c=1}^{C}\lambda \psi_{\text{kc}}\\ \mathrm{s.t.} & \Theta \geq 0,\Psi \geq 0, \end{array}\right) \end{array}$$

where Θ ≥ 0 and Ψ ≥ 0 mean each element *θ*_*ik*_ ≥ 0 and *ψ*_*kc*_ ≥ 0.

#### Parameter estimation

To solve the nonnegative constrained optimization problem, we use the multiplicative updating rules, which have a good compromise between speed and ease of implementation, to alternately update the model parameters Θ and Ψ [44]. We obtain the following two updating formulae for Θ and Ψ, respectively:

(11) $$\begin{array}{l} \theta_{\text{ik}}\leftarrow \theta_{\text{ik}}\frac{S_{i}\overset{C}{\underset{c=1}{\sum }}\frac{F_{\text{ic}}\psi_{\text{kc}}}{1-\exp\left(-\sum_{k=1}^{K}\theta_{\text{ik}}\psi_{\text{kc}}\right)}+\overset{N}{\underset{j=1}{\sum }}\frac{A_{\text{ij}}\theta_{\text{jk}}}{1-\exp\left(-\sum_{k=1}^{K}\theta_{\text{ik}}\theta_{\text{jk}}\right)}}{S_{i}\overset{C}{\underset{c=1}{\sum }}\psi_{\text{kc}}+\overset{N}{\underset{j=1}{\sum }}\theta_{\text{jk}}+\lambda }, \end{array}$$

and

(12) $$\psi_{\text{kc}}\leftarrow \psi_{\text{kc}}\frac{\overset{N}{\underset{i=1}{\sum }}S_{i}\frac{F_{\text{ic}}}{1-\exp\left(-\sum_{k=1}^{K}\theta_{\text{ik}}\psi_{\text{kc}}\right)}\theta_{\text{ik}}}{\overset{N}{\underset{i=1}{\sum }}S_{i}\theta_{\text{ik}}+\lambda }.$$

Due to the limitation in space, we describe the details of the two updating formulae in Additional file 1.

Once Θ and Ψ are initialized, we update them according to Equations (11) and (12) alternately until a stopping criterion has been satisfied. Since the objective function in Equation (10) is not convex, the final estimators of Θ and Ψ depend on their initial values. To mitigate the risk of local minimization to some extend, we repeat the entire updating procedure 100 times with random restarts and choose the result that gives the lowest value of the objective function as the final estimator. In our implementation, the iteration process is conducted until the relative change in objective value is less than 10^-6^. To avoid the case that this process converges too slowly and requires excessive computing time, we also stop it if the number of iterations reaches 400.

### Protein complex detection

After estimating Θ and Ψ, we still need to determine whether protein *i* belongs to complex *k* according to ${\hat{\theta }}_{\text{ik}}$. To this end, the rows of $\hat{\Theta }$ are normalized first such that $\sum_{k=1}^{K}{\hat{\theta }}_{\text{ik}}=1$. In effect, ${\hat{\theta }}_{\text{ik}}$ now represents the fraction by which protein *i* belongs to complex *k*. For a protein *i*, if ${\hat{\theta }}_{\text{ik}}=0$ (or <10^-16^) over all *k* before normalizing, we set ${\hat{\theta }}_{\text{ik}}=0$ during the normalization process. We then ignore the membership of protein *i* in complex *k* if ${\hat{\theta }}_{\text{ik}}$ is below a given threshold *τ*; otherwise, we regard protein *i* as belonging to complex *k*:

(13) $$\theta_{\text{ik}}^{⋆}=\left\{\begin{array}{ll} 1, & \text{if}\;{\hat{\theta }}_{\text{ik}}\geq \tau ,\\ 0, & \text{if}\;{\hat{\theta }}_{\text{ik}}<\tau . \end{array}\right)$$

Here $\Theta^{⋆}=[\theta_{\text{ik}}^{⋆}]$ is the protein-complex membership indication matrix in which $\theta_{\text{ik}}^{⋆}=1$ represents protein *i* is in the detected complex *k* and $\theta_{\text{ik}}^{⋆}=0$ represents protein *i* is not in complex *k*. We set *τ* = 0.2 experimentally such that a protein can not belong to more than 5 predicted complexes in our algorithm. Due to local minimization, a detected complex candidate may be composed of several isolated subnetworks. In this case, each connected subnetwork is regarded as a complex. We discard detected complexes which include less than three proteins.

**Figure 3:**
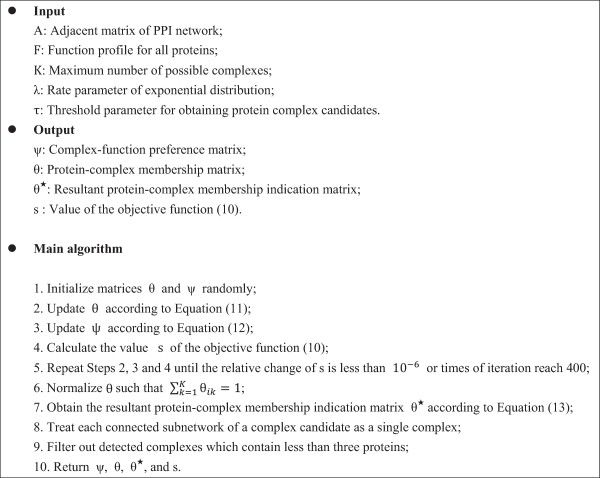
The algorithm of detecting protein complexes using GMFTP.

One issue in detecting complexes using GMFTP is to determine the number of complexes, *K*. That is because we usually do not have any prior knowledge about the number of complexes in real-world situations. Fortunately, we have used an exponential distribution prior over each element *θ*_*ik*_ and *ψ*_*kc*_, which makes the estimators $\hat{\Theta }$ and $\hat{\Psi }$ to be sparse and filters out the redundant complexes. Therefore, we can fit our model with a larger value of *K* as it is able to determine the number of complexes adaptively. In practice, a large number of proteins remain functionally uncharacterized. In order to prevent the negative impact of these unannotated proteins, we set *S*_*i*_ = 0 if the functional profile of protein *i* is not available, and *S*_*i*_ = 1 otherwise. The procedure of identifying protein complexes using GMFTP is illustrated in Figure 3.

## Results

### Data sets and evaluation methods

Two experimental yeast PPI data sets [4,5], a combined computational interaction map [45], the yeast interactions derived from DIP ( [46]) and the ones derived from BioGRID [47] are used to test the performance. We refer to them as Gavin, Krogan, Collins, DIP and BioGRID data sets. The Krogan data set is used as two variants: the core data set (referred to as Krogan core) and the extended data set (referred to as Krogan extended). The Collins, Gavin, Krogan core and Krogan extended data sets include edge weights. We derive two variants of these four networks: weighted version which includes the weights and unweighted version which ignores the weights. As DIP (version April 6, 2013) and BioGRID (version 3.1.77) provide weights for only a low proportion of the interactions, we treat them as unweighted, following the method in [17]. The Gene Ontology (April 6, 2013) is used as the data source of functional properties [48]. Four categories of functional profiles (BP, CC, MF and total) are derived from the annotations of the three individual subontologies (biological process, cellular component, and molecular function) and the comprehensive annotation which concatenates that of all the three subontologies. The gold standards of yeast protein complexes are derived from CYC2008 [19] and SGD [49]. For details, see Additional file 1.

We use four independent quality criteria, accuracy (ACC) [3], fraction of matched complexes (FRAC), maximum matching ratio (MMR) [17] and precision-recall score (PR) [40], to evaluate the detected complexes. The four metrics have complementary strengths since they evaluate the performance from different perspectives. Due to the fact that the gold standard complexes are incomplete, we also test the functional homogeneity of predicted complexes in a similar way to [17] (Additional file 1).

### Effect of parameters

GMFTP includes two parameters which need to be tuned: *K* and *λ*. As discussed above, we can use a value of *K* that is higher than the real number by introducing a sparse prior. We therefore set *K* = 1000 for all the six data sets. Next, we focus on examining the influence of *λ* which is the hyperparameter of prior distribution. We run GMFTP with various values of *λ* (*λ* ∈ {2^-3^,2^-2^,…,2^6^}) and evaluate the quality of predicted complexes by matching them with the reference complexes.

For each PPI network and each category of functional profile, the ACC and PR scores are used to test whether *λ* has an effect on the performance. Overall, GMFTP obtains competitive ACC scores when *λ*∈ [2^-3^,2^3^] and optimal PR scores when *λ*∈ [2^2^,2^4^] for both the two gold standards (Figures S2–S7 in Additional file 1). We also test how the parameter affects the number of predicted complexes and covered proteins. The number of predicted complexes and the number of proteins clustered into corresponding complexes decrease with increasing *λ* (Figures S2–S7 in Additional file 1), which shows that *λ* is able to control the sparsity of our model. An example which illustrates how *λ* influences the number of detected complexes via merging small complexes into larger ones is shown in Figure S8 (in Additional file 1). Overall, we find that GMFTP has a competitive performance when *λ* = 4 and other optimized values may improve further the performance in some cases. To avoid evaluation bias and overestimation of the performance, we do not tune the parameter to a particular dataset and set *λ* to 4 as the default value in the following experiments.

### Effect of functional property

To investigate the benefit brought by incorporating functional information into complex detection process, we compare the complexes predicted by GMFTP using only the PPI network to those using both the PPI network and the four categories of functional profiles. For the case of using only the PPI network, we set *S*_*i*_ = 0 for all proteins and *F* as a zero matrix with size *N* × *K*. For brevity, we refer to the five cases as PPI only, PPI+BP, PPI+CC, PPI+MF and PPI+total, respectively.

**Figure 4:**
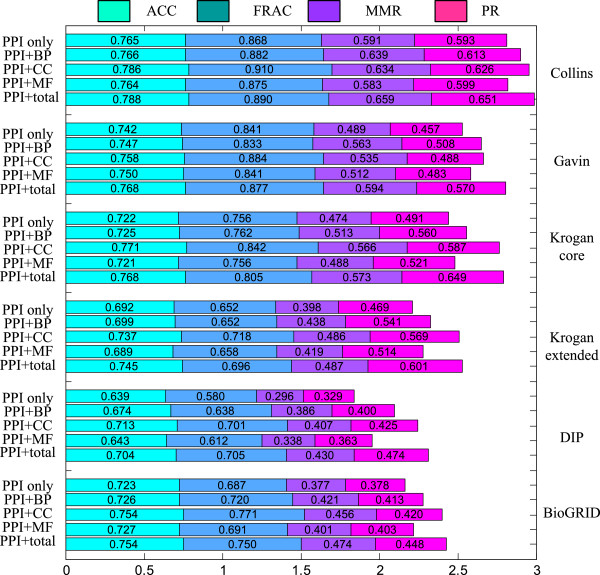
**Performance of GMFTP using different functional properties with respect to the CYC2008 gold standard.** The total height of each bar is the value of the composite scores of four metrics (ACC, FRAC, MMR and PR) for a given functional property on a given network. Larger scores are better.

For each case, the detected complexes are evaluated using the ACC, FRAC, MMR and PR scores with respect to the CYC2008 and SGD complexes (Figure 4, Figure S9 in Additional file 1). The PPI network combined with all the four categories of functional profiles works better than the PPI network alone, which shows that incorporating functional property into GMFTP is always able to improve the quality of detected complexes. In general, the results of CC property outperform those of BP and MF properties. This is partly because the functional profile of CC subontology may actually give some hints as to what complex(es) a protein may belong to. The BP functional profile usually performs a little better than the MF functional profile. This may in part be due to the richer annotations in the BP subontology. We also observe that the total functional profile generally performs better than the other three individual functional profiles except several results using the SGD gold standard. This demonstrates that the GO annotations of the three orthogonal subontologies have complimentary strength in capturing functional homogeneity of complexes, and that merging them is able to improve the performance.To understand how the functional properties help to improve the performance, let us go back to the example illustrated in Figure 1. Protein YML007W does not participate in SAGA complex but interacts with a total of seven proteins in this complex. GMFTP using only the topological property incorrectly clusters it into this complex (Figure 1(a)). Due to the fewer interactions with the core members of transcription factor TFIID complex, protein YPL129W is neglected when using only the PPI network. From Figure 1(b), we can find that protein YML007W does not associate with functions which are frequently associated with the members of SAGA complex (e.g., GO:0003712 and GO:0016573), thus it is filtered out when the functional information is taken into account. Since protein YPL129W shares common functions (e.g., GO:0001075 and GO:0051123) with the members of transcription factor TFIID complex, it is correctly grouped into this complex. Since protein YER164W neither interacts nor has many similar functions with the other members of SAGA complex, it cannot be recovered by our model correctly.

### Comparison with previous approaches using only topological property

Since most previous approaches detect complexes based solely on the PPI network, we concentrate on testing the effectiveness of GMFTP using only the topological property first. We compare it to a representative set of approaches: AP [11], CFinder [50], ClusterONE [17], Linkcomm [38], MCL [9], MCODE [10], MINE [51], SPICi [12] and SR-MCL [32]. For the four algorithms (AP, ClusterONE, MCL and SPICi) which can handle weights, we implement them on both the weighted and the unweighted versions of the four networks (Collins, Gavin, Krogan core and Krogan extended) which include edge weights. For each algorithm, except ClusterONE, Linkcomm and SR-MCL for which we use the default parameters as suggested by the authors, the parameters are deliberately selected in a similar way to [17]. The details are listed in Additional file 1. For all compared approaches, like GMFTP, we exclude complex candidates with size less than three. For GMFTP, we set *F* as a zero matrix and *S*_*i*_ = 0 for all proteins in this experiment. We do not tune the parameters of GMFTP and set *K* = 1000, *λ* = 4 for all datasets.

Table 1 shows the overall comparative results on the unweighted networks using the CYC2008 gold standard. We find the relative performances of these approaches change according to the topological properties of the networks under consideration and the evaluation metrics we use. CFinder and MCODE tend to identify fewer complexes; Linkcomm and SR-MCL detect more complexes. The other six methods usually perform as a compromise between the two extreme cases. When we only consider how well the gold standards are recovered by the predicted complexes (quantified using FRAC and MMR), Linkcomm and SR-MCL achieves better performance than the other methods partially because they detect more complexes. When we pay attention not only to how well the reference sets are recovered by the predicted complexes but to how well the predicted complexes match to the reference sets (quantified using ACC and PR), GMFTP outperforms the previous nine approaches with a few exceptions. Furthermore, GMFTP also gets competitive FRAC and MMR scores except the two extreme cases (Linkcomm and SR-MCL). Similar results are also observed using the SGD reference complexes (Additional file 2). When we implement ClusterONE on the weighted version of the four networks (Collins, Gavin, Krogan core and Krogan extended), it gets higher FRAC, MMR and ACC scores than GMFTP in some cases (Additional file 2). Due to the competitive performance of GMFTP on the unweighted versions, we may therefore conjecture that the better performance of ClusterONE using weights comes from the ability to take weights into account, and the competitive performance of GMFTP on the unweighted networks may be due to a fundamentally different underlying algorithm.

**Table 1:** Benchmark results using solely the unweighted PPI network with respect to the CYC2008 gold standard

	**Algorithm**	**Coverage**	**# Complexes**	**FRAC**	**MMR**	**ACC**	**PR**
	GMFTP	1168	179	0.868	0.591	0.765	*0.593*
	AP	1363	207	0.697	0.785	0.497	0.444
	CFinder	1161	114	0.653	0.439	0.693	0.440
	ClusterONE	1293	203	0.847	0.571	0.775	0.564
	Linkcomm	1126	407	*0.903*	*0.646*	0.744	0.456
Collins	MCL	1178	187	0.840	0.537	*0.779*	0.529
	MCODE	853	115	0.743	0.496	0.730	*0.593*
	MINE	1101	138	0.771	0.499	0.756	0.547
	SPICi	958	124	0.708	0.448	0.728	0.570
	SR-MCL	1304	337	0.875	0.625	0.755	0.481
	GMFTP	1464	271	0.841	0.489	*0.742*	0.457
	AP	1815	274	0.667	0.659	0.346	0.310
	CFinder	1158	137	0.638	0.378	0.701	0.424
	ClusterONE	1624	294	0.783	0.449	0.725	0.391
	Linkcomm	1381	604	*0.870*	*0.548*	0.703	0.372
Gavin	MCL	1301	240	0.696	0.421	0.713	0.422
	MCODE	899	155	0.710	0.438	0.685	*0.492*
	MINE	1242	212	0.804	0.454	0.710	0.436
	SPICi	1008	184	0.746	0.434	0.697	0.478
	SR-MCL	1750	735	0.819	0.539	0.701	0.327
	GMFTP	1244	270	0.756	0.474	*0.722*	*0.491*
	AP	2506	391	0.575	0.433	0.242	0.182
	CFinder	1143	115	0.433	0.281	0.555	0.268
	ClusterONE	2044	539	0.720	0.431	0.708	0.326
	Linkcomm	962	425	0.701	0.460	0.675	0.428
Krogan core	MCL	1933	388	0.671	0.377	0.691	0.299
	MCODE	640	95	0.463	0.268	0.583	0.406
	MINE	937	157	0.616	0.359	0.664	0.450
	SPICi	1249	224	0.628	0.356	0.689	0.409
	SR-MCL	2585	1833	*0.884*	*0.575*	0.686	0.197
	GMFTP	1197	265	0.652	0.398	*0.692*	*0.469*
	AP	3522	461	0.461	0.232	0.117	0.096
	CFinder	914	88	0.287	0.172	0.543	0.277
	ClusterONE	1114	239	0.481	0.296	0.633	0.407
	Linkcomm	1925	998	0.652	0.424	0.687	0.317
Krogan extended	MCL	2973	531	0.503	0.254	0.636	0.190
	MCODE	619	84	0.343	0.188	0.506	0.345
	MINE	902	162	0.564	0.316	0.650	0.451
	SPICi	1584	295	0.525	0.258	0.645	0.311
	SR-MCL	3637	2644	*0.702*	*0.431*	0.617	0.154
	GMFTP	1705	376	0.580	0.296	*0.639*	0.329
	AP	4662	517	0.441	0.219	0.091	0.086
	CFinder	635	75	0.263	0.119	0.453	0.297
	ClusterONE	1402	346	0.429	0.227	0.554	0.280
	Linkcomm	3396	1829	*0.630*	*0.386*	0.629	0.203
DIP	MCL	4007	609	0.451	0.234	0.628	0.173
	MCODE	540	95	0.210	0.108	0.402	0.211
	MINE	1135	260	0.536	0.268	0.585	*0.333*
	SPICi	2103	403	0.455	0.228	0.583	0.245
	SR-MCL	4825	3222	0.674	0.376	0.583	0.141
	GMFTP	2456	434	*0.687*	0.377	*0.723*	*0.378*
	AP	5632	206	0.316	0.064	0.027	0.044
	CFinder	1729	110	0.220	0.127	0.512	0.186
	ClusterONE	2580	473	0.610	0.318	0.683	0.325
	Linkcomm	4119	4446	0.678	*0.459*	0.701	0.243
BioGRID	MCL	3652	335	0.314	0.158	0.520	0.126
	MCODE	1087	136	0.297	0.154	0.514	0.294
	MINE	2414	409	0.576	0.308	0.663	0.304
	SPICi	2756	501	0.483	0.261	0.652	0.281
	SR-MCL	5593	1097	0.496	0.273	0.594	0.143

We also compare the functional homogeneity of predicted complexes through calculating the enrichment of Gene Ontology functions. Since Linkcomm and SR-MCL get better FRAC and MMR scores than GMFTP, we focus on comparing GMFTP with them. Table 2 lists the number (and percentage) of the identified complexes whose P-values falls within P-values < E-15, [E-15, E-10], [E-10, E-5], [E-5, 1]. Note that here the P-value of each identified complex is calculated using the total GO functions of all the three subontologies (BP, CC and MF), and the results of each subontology are listed in Additional file 3. There are more complexes detected by GMFTP than by the other two methods with P-value less than E-15, E-10, or E-5 in terms of percentage. This indicates that even though Linkcomm and SR-MCL detect more complexes such that they can recall the reference complexes well, they also detect more complexes which are less functional significant. In summary, Linkcomm and SR-MCL have more competitive recall ratio; but GMFTP has a good compromise between recall and precision.

**Table 2:** Functional enrichment of the complexes detected using only the unweighted PPI network

**Network**	**Algorithm**	**<E(-15)**	**E(-15) to E(-10)**	**E(-10) to E(-5)**	**E(-5) to 1**
Collins	GMFTP	33 (18.4%)	24 (13.4%)	60 (33.5%)	62 (34.6%)
	Linkcomm	53 (13.0%)	59 (14.5%)	109 (26.8%)	186 (45.7%)
	SR-MCL	38 (11.3%)	36 (10.7%)	92 (27.3%)	171 (50.7%)
Gavin	GMFTP	29 (10.7%)	20 (7.4%)	54 (19.9%)	168 (62.0%)
	Linkcomm	29 (4.8%)	34 (5.6%)	112 (18.5%)	429 (71.0%)
	SR-MCL	49 (6.7%)	29 (3.9%)	135 (18.4%)	522 (71.0%)
Krogan core	GMFTP	28 (10.4%)	22 (8.1%)	63 (23.3%)	157 (58.1%)
	Linkcomm	24 (5.6%)	30 (7.1%)	114 (26.8%)	257 (60.5%)
	SR-MCL	80 (4.4%)	70 (3.8%)	264 (14.4%)	1419 (77.4%)
Krogan extended	GMFTP	29 (10.9%)	19 (7.2%)	57 (21.5%)	160 (60.4%)
	Linkcomm	30 (3.0%)	41 (4.1%)	158 (15.8%)	769 (77.1%)
	SR-MCL	135 (5.1%)	86 (3.3%)	259 (9.8%)	2164 (81.8%)
DIP	GMFTP	36 (9.6%)	29 (7.7%)	68 (18.1%)	242 (64.5%)
	Linkcomm	44 (2.4%)	63 (3.4%)	323 (17.7%)	1398 (76.5%)
	SR-MCL	174 (5.4%)	117 (3.6%)	398 (12.3%)	2533 (78.6%)
BioGRID	GMFTP	66 (15.2%)	38 (8.8%)	113 (26.0%)	217 (50.0%)
	Linkcomm	217 (4.9%)	254 (5.7%)	1026 (23.1%)	2949 (66.3%)
	SR-MCL	166 (15.1%)	77 (7.0%)	210 (19.2%)	643 (58.7%)

### Comparison with previous approaches using both functional and topological properties

To evaluate the advantage of GMFTP in incorporating functional annotation into complex detection process, we compare its results with those of other approaches which also take functional property into consideration. A popular framework on this topic can be divided into two steps: to weight the strengths of interactions using some semantic similarity measures, and then to detect complexes from the weighted PPI networks using some graph clustering algorithms [22-25]. The main difference between them lies in the different similarity measures and clustering algorithms they use. Since there is no public software available for these approaches, we design a heuristic comparison. We employ three widely used measures Jiang ( [52], Kappa [53] and Lin [54]) to weight the PPI network and apply four algorithms (AP, ClusterONE, MCL and SPICi) which can handle weights to detect complexes. The package csbl.go [55] is used to calculate the similarities between proteins, and the weights of interactions which involve unannotated proteins are set to 1. The parameter settings of the clustering algorithms are presented in Additional file 1. We also compare GMFTP to COAN [34] which considers GO slim annotations by constructing an ontology augmented network.

Table 3 presents the comparative performance with ClusterONE using the total GO annotation with respect to the CYC2008 reference. The results of the three individual subontologies (BP, CC and MF), the other four clustering algorithms (AP, COAN, MCL and SPICi) and the SGD gold standard are reported in Additional file 2. For each clustering algorithm (AP, ClusterONE, MCL and SPICi), the performance differs a little with different GO similarity measures; and for each semantic measure (Jiang, Kappa and Lin), the relative performance changes depending on the clustering algorithm and the PPI network under consideration. We also find that the relative performance of each clustering algorithm and each semantic measure depends on the functional property of each subontology individually, which indicates that there is no single clustering algorithm and semantic measure that can dominate the rest in all cases. Overall, GMFTP and ClusterONE are competitive. In some cases, ClusterONE with deliberately selected semantic measure may obtain higher ACC scores than GMFTP. However, GMFTP outperforms ClusterONE in terms of the MMR and PR scores. For the Collins, Gavin and BioGRID networks, SPICi achievers better performance than GMFTP using the PR score under some circumstances, but GMFTP is superior to SPICi using the other three evaluation scores. What is more, GMFTP achieves the best MMR score which is a new evaluation measure recommended in [17] in most cases. These results demonstrate that GMFTP is an effective approach that can make full use of the topological and functional properties for protein complex identification.

**Table 3:** Benchmark results using both the PPI network and the total GO annotation with respect to the CYC2008 gold standard

**Network**	**Algorithm**	**GO sim**	**Coverage**	**# Complexes**	**FRAC**	**MMR**	**ACC**	**PR**
Collins	GMFTP	–	1085	188	*0.890*	*0.659*	*0.788*	*0.651*
		Jiang	1210	169	0.868	0.575	0.784	0.579
	ClusterONE	Kappa	1130	161	0.840	0.573	0.770	0.597
		Lin	1255	172	0.854	0.561	0.784	0.613
Gavin	GMFTP	–	1122	208	*0.877*	*0.594*	*0.768*	*0.577*
		Jiang	1298	224	0.833	0.549	*0.768*	0.496
	ClusterONE	Kappa	1218	217	0.783	0.511	0.760	0.485
		Lin	1461	253	0.804	0.514	0.757	0.449
Krogan core	GMFTP	–	1218	252	0.805	*0.573*	*0.768*	*0.649*
		Jiang	1516	341	*0.847*	0.540	0.756	0.445
	ClusterONE	Kappa	1322	319	0.810	0.522	0.736	0.470
		Lin	1813	464	0.841	0.517	0.766	0.379
Krogan extended	GMFTP	–	1062	216	0.696	*0.487*	*0.745*	*0.601*
		Jiang	2021	503	*0.724*	0.435	*0.745*	0.342
	ClusterONE	Kappa	1722	482	0.680	0.410	0.733	0.344
		Lin	2458	752	0.713	0.425	0.730	0.281
DIP	GMFTP	–	1492	306	0.705	*0.430*	0.704	*0.474*
		Jiang	2910	733	*0.741*	0.406	*0.714*	0.299
	ClusterONE	Kappa	2487	748	0.679	0.398	0.682	0.308
		Lin	3285	921	0.701	0.359	0.700	0.248
BioGRID	GMFTP	–	2283	413	*0.750*	*0.474*	0.754	*0.448*
		Jiang	3789	881	0.665	0.377	*0.759*	0.260
	ClusterONE	Kappa	3303	889	0.691	0.398	0.717	0.283
		Lin	4208	1073	0.602	0.334	0.755	0.227

### Detecting multifunctional proteins

It is well known that a protein may carry out different functions in different complexes. A desirable approach to complex detection therefore should be able to accommodate proteins that belong to more than one complex. Due to the absence of a reference set of bona fide multifunctional proteins, it is impractical to compare different approaches at this job directly. We resort to test how well the set of multi-group proteins predicted by GMFTP matches with those of the other methods which also handle overlaps (CFinder, ClusterONE, Linkcomm, MINE and SR-MCL) and the two gold standards (CYC2008 and SGD). For GMFTP, we concentrate on the results of two cases (PPI only and PPI+total). For ClusterONE, we use the results of the two versions (weighted and unweighted) of networks. A protein is regarded as a multi-group protein if it belongs to more than one predicted (or reference) complex, and it is a mono-group protein if it belongs only to one predicted (or reference) complex. Overall, the multi-group proteins recovered by our model significantly (hypergeometric test, P-value ≤ 0.01) overlap with those of the other approaches and the gold standards (Additional file 4).

**Figure 5:**
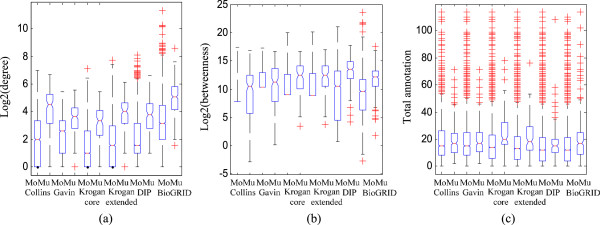
**Topological and functional features of mono (Mo)- and multi (Mu)-group proteins detected by GMFTP.** This result is obtained using the PPI network and the total GO annotation. For each feature, the distributions of mono- and multi-grouped proteins are represented by boxplots (line = median). **(a)** Degree. **(b)** Betweenness. **(c)** Number of annotated functions of all the three subontologies.

In a similar manner to [18], we further focus on testing whether topological and functional features can distinguish multi- and mono-group proteins identified by GMFTP. Here we concentrate on the results detected using the PPI network and the total GO annotation for its competitive performance, of which the general statistics are listed in Additional file 4. From Figure 5, we observe that the multi-group proteins have, on average, a higher degree, a higher node betweenness and a higher number of annotated GO functions. This is also true for the number of functional annotations of the three individual subontologies except the MF ontology (Figure S10 in Additional file 1). We implement Wilcoxon rank-sum test to assess whether the differences of distributions of the topological and functional features between multi- and mono-clustered proteins are statistically significant. The results presented in Additional file 4 show that the differences are significant (P-value ≤ 0.01) in most cases. The multi-group proteins recovered by GMFTP are therefore more central in the network and are involved more biological functions.

## Discussion

The developments of high-throughput experimental techniques and computational methods for delineating protein-protein interactions and predicting protein functions have produced rich interaction and functional knowledge of proteins. Recently, a great deal of research works have tried to group proteins into complexes in a given PPI network. However, the performances of the approaches which use the topological property alone are limited not only for the poor quality of the underlying PPI network but also for the negligence of other available information such as functional profile.

In our opinion, both topological and functional properties are meaningful and important for predicting protein complexes. We therefore develop a new algorithm which makes full use of them. Unlike previous approaches, we consider an alternative view and propose a probabilistic model-based approach to combine these two types of properties in a natural and principled manner. Our method can avoid the choice of semantic measures and naturally deal with overlaps. Owing to the superior performance and sound theoretical principle of GMFTP, we hope that our work can attract more attention to model-based methods for complex detection. Although generative model have been applied to study PPI networks, our model is different from the previous ones since most of them focus only on the generative process of the network structure. As we know, our model is one of the first to take the generative process of the functional profile into account.

One problem with considering functional property is that the improvement of performance depends on the quality and completeness of functional annotations of the database. It is well known that functional information is not always obtainable in practice [40]. From Equation (11), the complex(es) into which an uncharacterized protein will be clustered is determined only by the topological structure, which means our model can adaptively handle the case where the protein is not functionally characterized. Since GO terms in the subontology of cellular component may provide some clues as to what complex(es) a protein may belong to, the function property derived from this subontology may introduce biases and overestimate the performance. However, the effectiveness of our model has also been investigated in the other two subontologies. In practical application, even if there may be some evaluation biases, we suggest combining the total GO annotations of all the three subontologies to form a comprehensive functional profile to improve the performance, which works similarly to the semi-supervised clustering in machine learning [56].

In general, it is time-consuming and difficult for model-based approaches to scale up. We now analyze the computational complexity in Equations (11) and (12). Each update of Θ takes *O*(*KN*(*N* + *C*)) times and update of Ψ takes *O*(*NKC*) times. Therefore, the total time cost of GMFTP is *O*(*KNT*(*N* + *C*)), where *T* is the number of iterations. Given that the real-world PPI networks and functional profiles are extremely sparse, the overall cost can be reduced to *O*(*KT*(*N* + *C* + *E* + *R*)), where *E* is the number of interactions and *R* is the number of functional associations (see Additional file 1). In the experiments, we implement the algorithm using Matlab in a workstation with Intel 4 CPU (3.40 GH × 4) and 16 GB RAM. Each update costs at most 3.25 seconds and the entire estimation takes less than 1300 seconds when we set the maximum number of iterations to 400. This means that even though our approach may be not as fast as some local network clustering algorithms (e.g., SPICi), the time cost is also affordable. In order to avoid local minimization, we repeat the updating process 100 times with random restarts. We acknowledge that this may be not a sufficient number of repetitions to ensure a global optimum solution and GMFTP would work better with more restarts. Instead of searching for the global minimization with millions of repetitions, we have paid attention to evaluate how the random initial conditions influence the stability of the results (see Additional file 1).

One perennial problem for model-based approaches is to select models, that is how to determine the value of parameter *λ* here. In statistics, several model selection strategies are available [43]. A simple and widely adopted strategy is the cross-validation procedure. However, this strategy may be not applicable in the task of network clustering since removing a predefined fraction of proteins (or interactions) from a PPI network would change the topological structure, which means adding noise rather than splitting the data set [17]. Another solution to this problem is to select model according to some model selection criteria such as Akaike information criterion and Bayesian information criterion. The performance of this type of strategies varies according to the choice of criteria. For simplicity and good performance, we first analyze how *λ* affects the performance and then set it to 4 in the comparative experiments. The model selection problem is left as an open research question in the future study.

Previous researches have shown that the quality of detected complexes could be improved if the weights of interactions are available [17]. Currently, our model is limited to unweighted networks and can be applied to weighted networks only after “binarizing” them due to the Bernoulli generative mechanism. In the future work, we will investigate the generative process of weighted networks to make full use of the valuable information of weights. In addition, the hierarchical relationships among GO terms are not used in our model. Intuitively, two proteins which share a low-level (or specific) GO function are more likely to belong to the common complex(es) than those which share a high-level (or general) GO function. It would be useful to incorporate the specificity of GO terms into our model and further to improve the performance.

## Conclusions

In this study, we have developed a new approach for protein complex detection based on a proposed generative model for protein-protein interaction network and protein functional profile. Experiment results on six yeast networks show the competitive performance of our method in the identification of both protein complexes and multifunctional proteins. The results also show the effect of protein functional property on complex detection, which suggests that the functional annotation information should be used if it is available.

## Competing interests

The authors declare that they have no competing interests.

## Authors’ contributions

XFZ, DQD, LOY and HY designed the method and conceived the study. XFZ and LOY implemented the method and performed the experiments. XFZ, DQD, LOY and HY wrote the paper. All authors read and approved the final manuscript.

## Supplementary Material

Additional file 1 **Supplementary figures and text.** This section provides the supplementary figures referred in the main text and some text which describes the parameter estimation method, the data sets we use, the evaluation methods we use, convergence and computational complexity analysis of the proposed model, effects of random restarts and parameter *K*, and parameter settings of compared algorithms. Click here for file

Additional file 2 **Benchmark results of comparative experiments.** This section provides the supplementary tables which describe the comparative results for the six datasets (Collins, Gavin, Krogan core, Krogan extended, DIP and BioGRID). Click here for file

Additional file 3 **Functional enrichment of the detected protein complexes.** We provide the functional enrichment analysis results of the complexes predicted by GMFTP, Linkcomm and SR-MCL using only the PPI network with respect to the three individual subontology (BP, MF, CC) in this section. Click here for file

Additional file 4 **Supplementary tables for the analysis of multifunctional proteins detection.** This file include supplementary tables which describe the general properties of multi-group proteins detected by various approaches, the statistical results of the complexes predicted by GMFTP using the PPI network and the total GO annotation, and P-value of Wilcoxon test of populations of topological and functional features of mono- and multi-grouped proteins detected by GMFTP. Click here for file
