# Supplementary material for: Detecting overlapping protein complexes based on a generative model with functional and topological properties
Source: BMC Bioinformatics. 2014 Jun 13;15:186. doi: 10.1186/1471-2105-15-186 (PMC4073817; doi:10.1186/1471-2105-15-186)
Supplement: Additional file 1 — Supplementary figures and text. This section provides the supplementary figures referred in the main text and some text which describes the parameter estimation method, the data sets we use, the evaluation methods we use, convergence and computational complexity analysis of the proposed model, effects of random restarts and parameter K, and parameter settings of compared algorithms. [file 1471-2105-15-186-S1.pdf]

Detecting overlapping protein complexes based on a generative model with functional and topological properties: Additional file 1

Xiao-Fei Zhang, Dao-Qing Dai, Le Ou-Yang and Hong Yan

Contents

1 Supplementary Figure **2**

2 Supplementary Text **12**

2.1 Model parameter estimation 12
2.2 Data sets 13
2.3 Evaluation methods 14
2.4 Convergence and computational complexity analysis 15
2.5 Effect of random restarts 17
2.6 Effect of K 17
2.7 Parameter settings of compared algorithms 17

1 Supplementary Figure

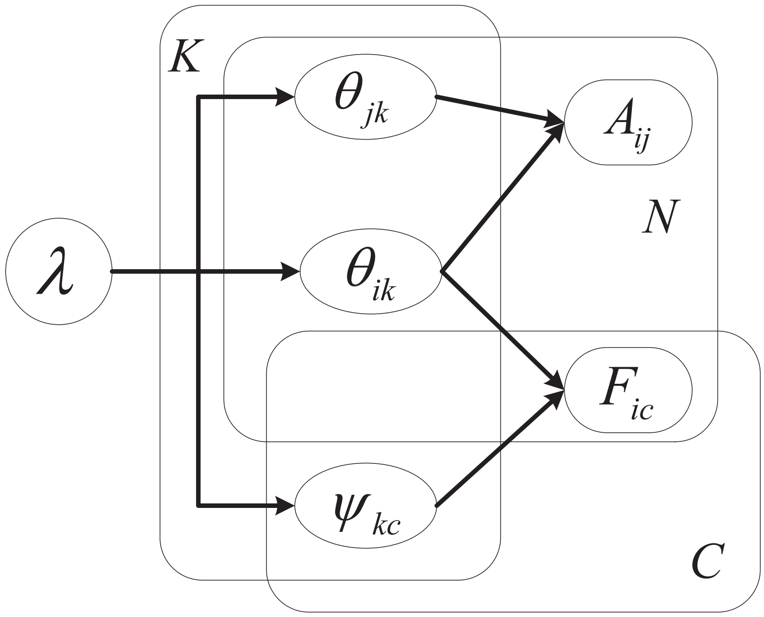

Figure S1: A directed graphical representation of the proposed model. The plate notation is used here. The plate is used to group random variables that repeat. The number of replicate is shown on the corner. $\{A_{ij}\}$ and $\{F_{ic}\}$ are observed variables; θ_{ik} and ψ_{kc} are latent variables; and λ is hyperparameter.

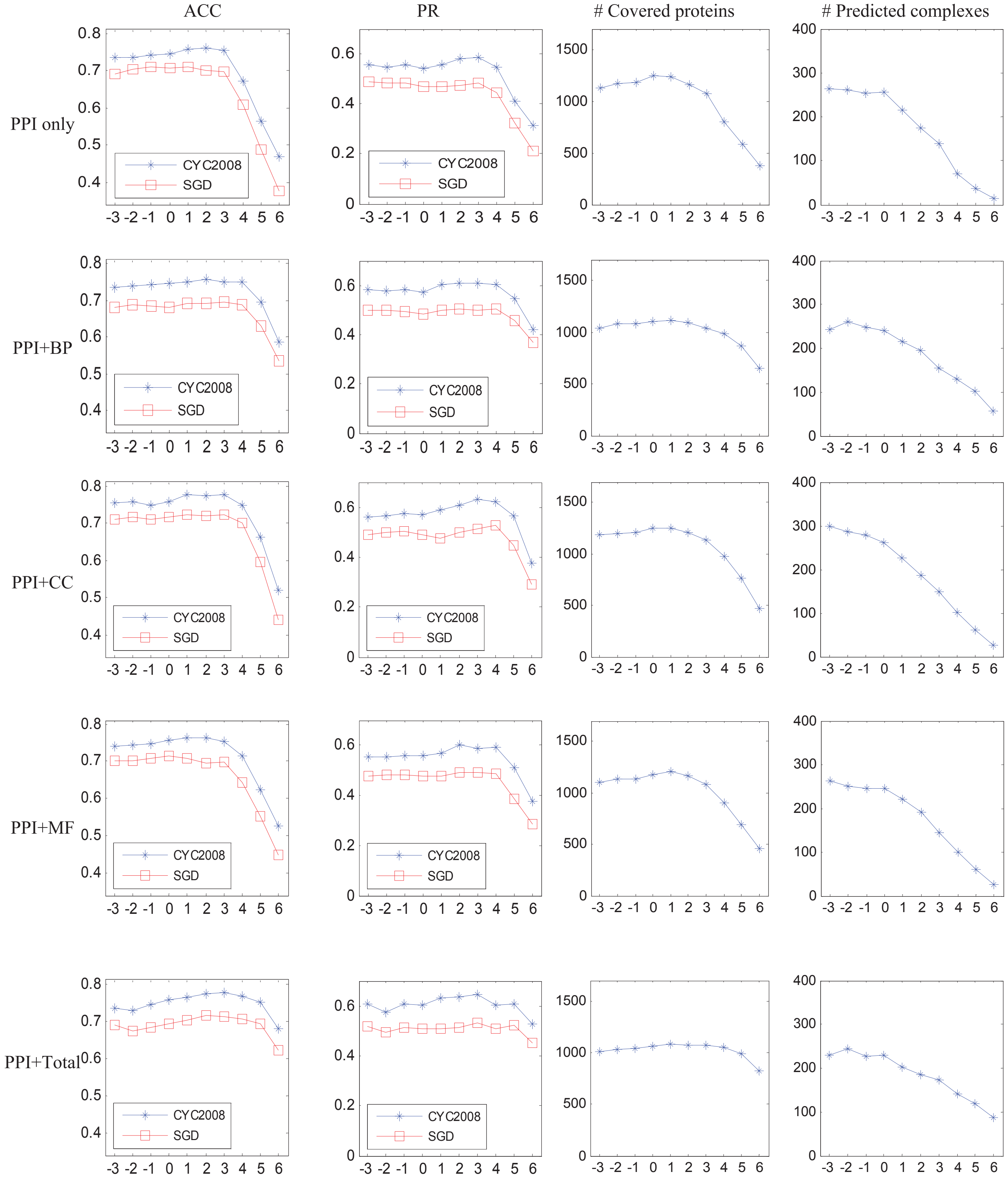

Figure S2: Performance of GMFTP with respect to different value of λ on the Collins network. From top to down, each row represents the results of the five categories of functional properties (PPI only, PPI+BP, PPI+CC, PPI+MF, PPI+total). From left to right, each column represents the results of the four criteria used to judge performance (ACC, PR, the number of covered protein and the number of detected complexes). For each figure, the x-axis denotes the value of $\log \lambda$, and y-axis denotes the value of corresponding evaluation criterion.

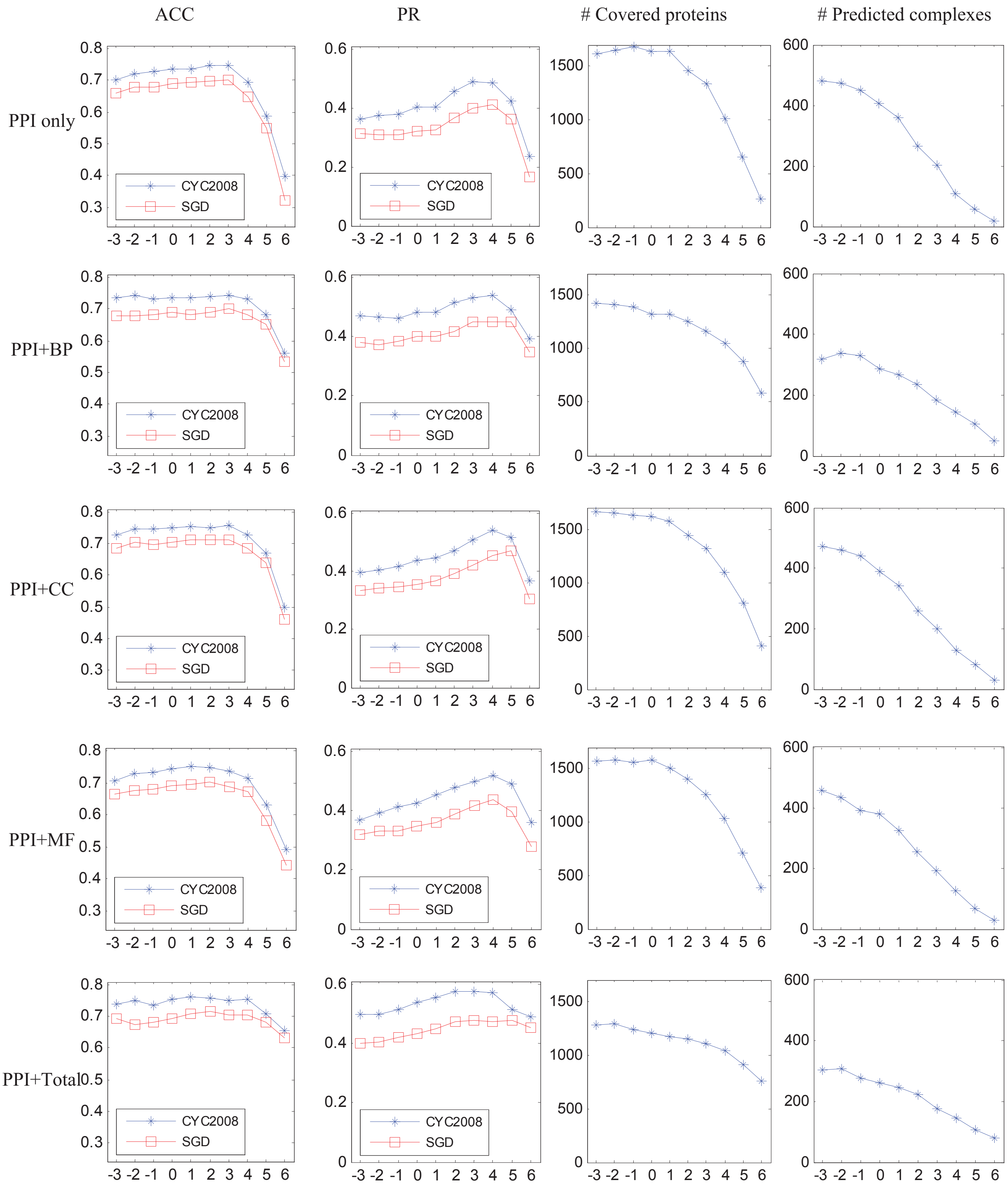

Figure S3: Performance of GMFTP with respect to different value of λ on the Gavin network. From top to down, each row represents the results of the five categories of functional properties (PPI only, PPI+BP, PPI+CC, PPI+MF, PPI+total). From left to right, each column represents the results of the four criteria used to judge performance (ACC, PR, the number of covered protein and the number of detected complexes). For each figure, the x-axis denotes the value of $\log \lambda$, and y-axis denotes the value of corresponding evaluation criterion.

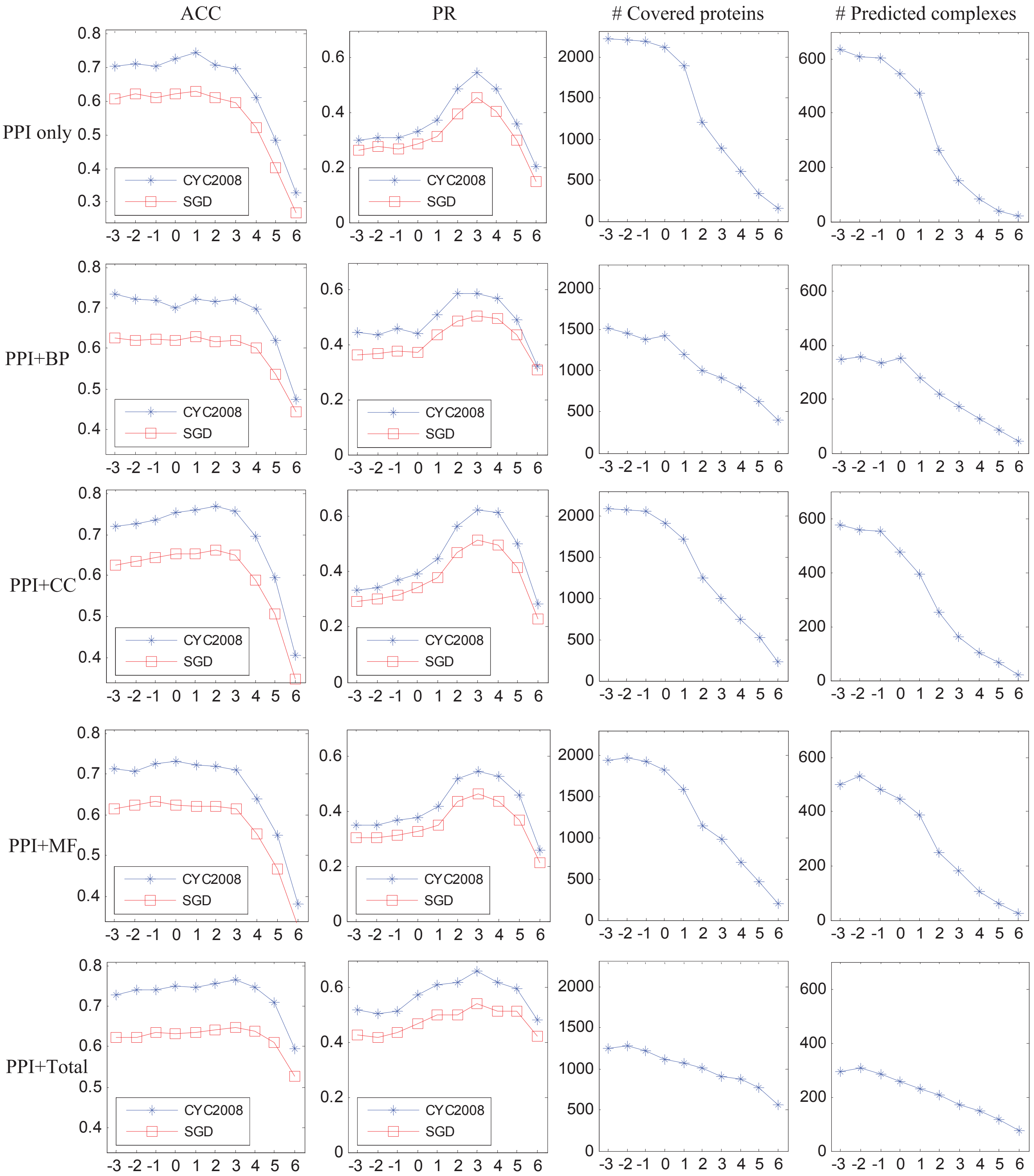

Figure S4: Performance of GMFTP with respect to different value of λ on the Krogan core network. From top to down, each row represents the results of the five categories of functional properties (PPI only, PPI+BP, PPI+CC, PPI+MF, PPI+total). From left to right, each column represents the results of the four criteria used to judge performance (ACC, PR, the number of covered protein and the number of detected complexes). For each figure, the x-axis denotes the value of $\log \lambda$, and y-axis denotes the value of corresponding evaluation criterion.

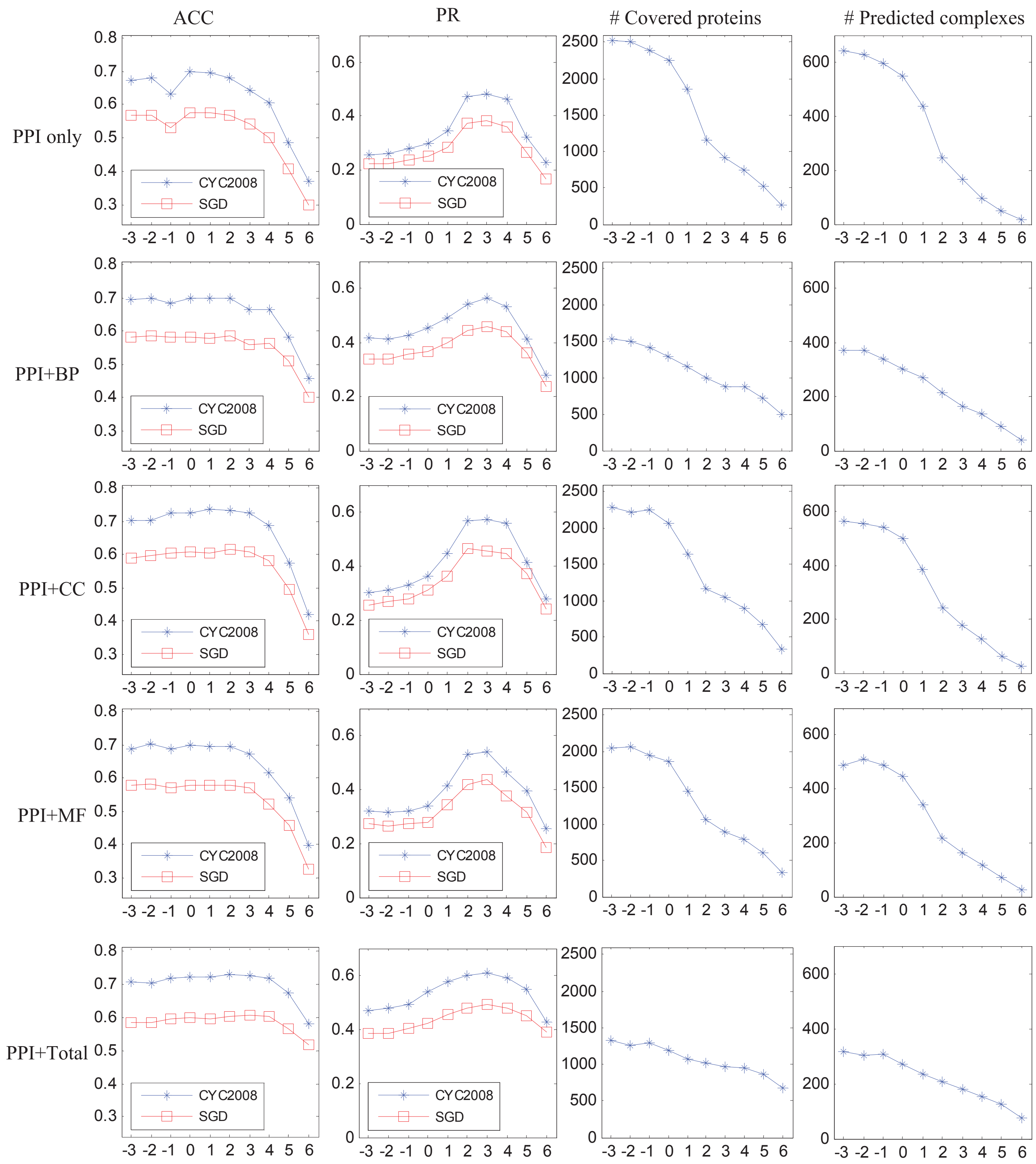

Figure S5: Performance of GMFTP with respect to different value of λ on the Krogan extended network. From top to down, each row represents the results of the five categories of functional properties (PPI only, PPI+BP, PPI+CC, PPI+MF, PPI+total). From left to right, each column represents the results of the four criteria used to judge performance (ACC, PR, the number of covered protein and the number of detected complexes). For each figure, the x-axis denotes the value of $\log \lambda$, and y-axis denotes the value of corresponding evaluation criterion.

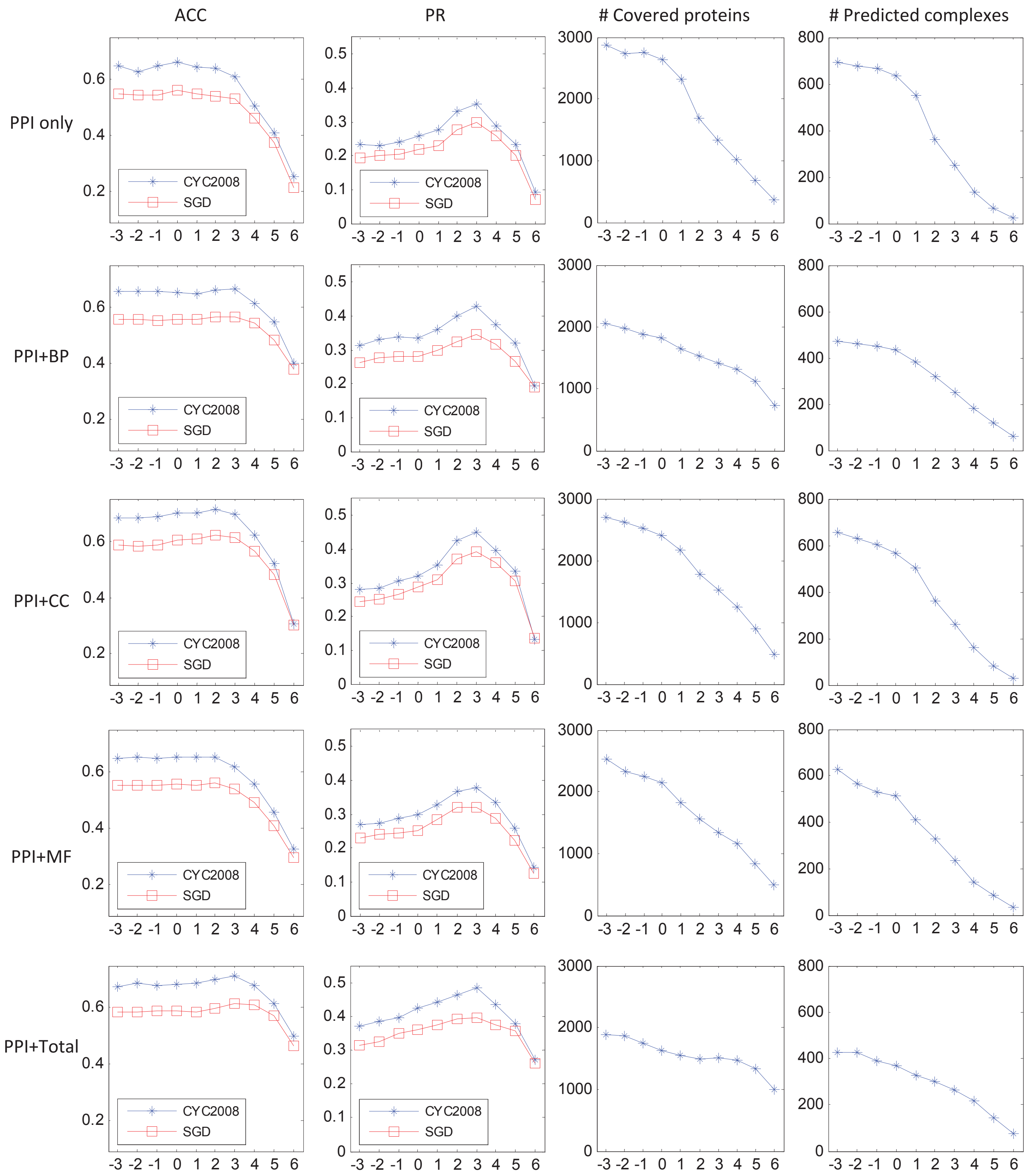

Figure S6: Performance of GMFTP with respect to different value of λ on the DIP network. From top to down, each row represents the results of the five categories of functional properties (PPI only, PPI+BP, PPI+CC, PPI+MF, PPI+total). From left to right, each column represents the results of the four criteria used to judge performance (ACC, PR, the number of covered protein and the number of detected complexes). For each figure, the x-axis denotes the value of $\log \lambda$, and y-axis denotes the value of corresponding evaluation criterion.

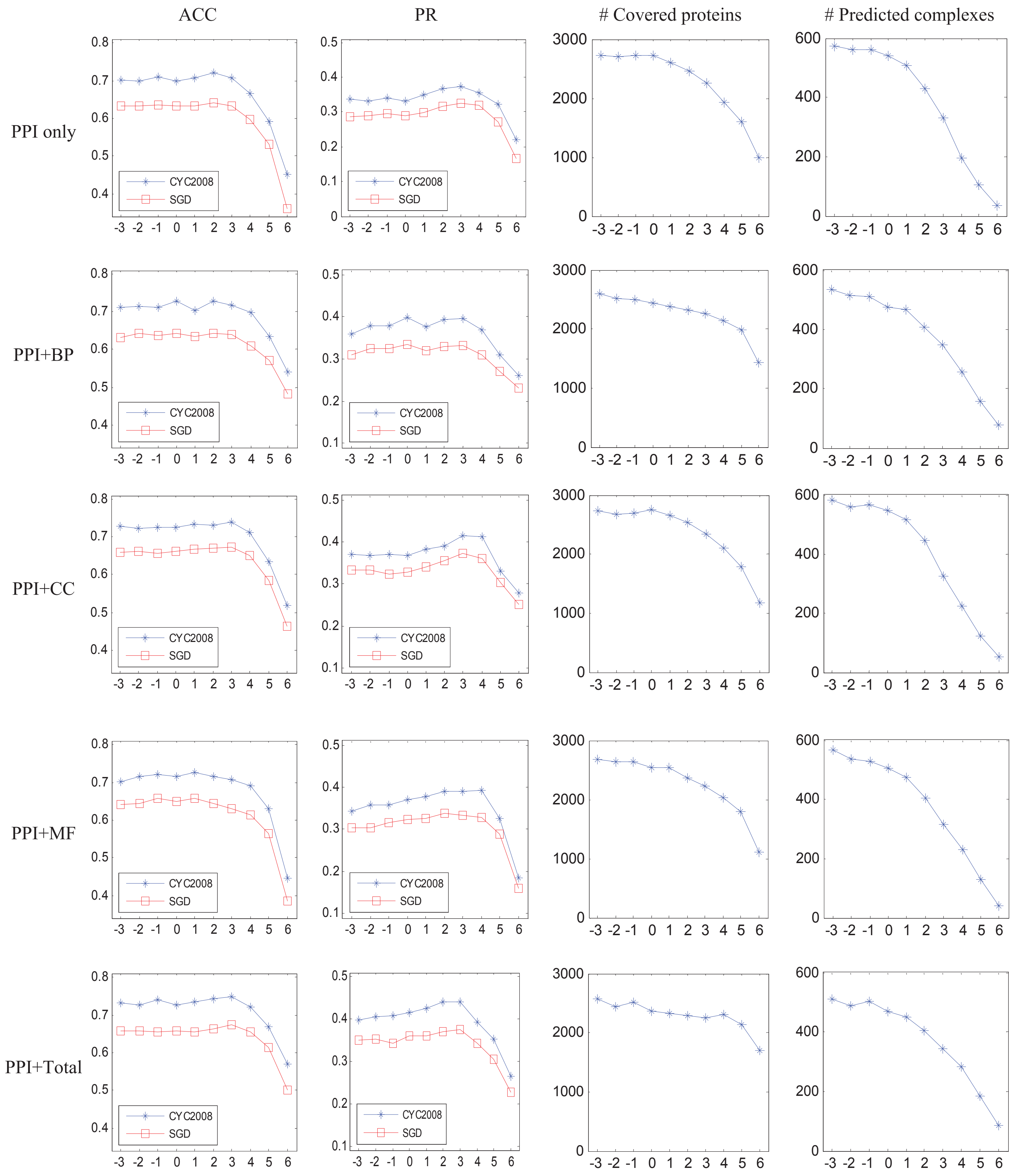

Figure S7: Performance of GMFTP with respect to different values of λ on the BioGRID network. From top to down, each row represents the results of the five categories of functional properties (PPI only, PPI+BP, PPI+CC, PPI+MF, PPI+total). From left to right, each column represents the results of the four criteria used to judge performance (ACC, PR, the number of covered protein and the number of detected complexes). For each figure, the x-axis denotes the value of $\log \lambda$, and y-axis denotes the value of corresponding evaluation criterion.

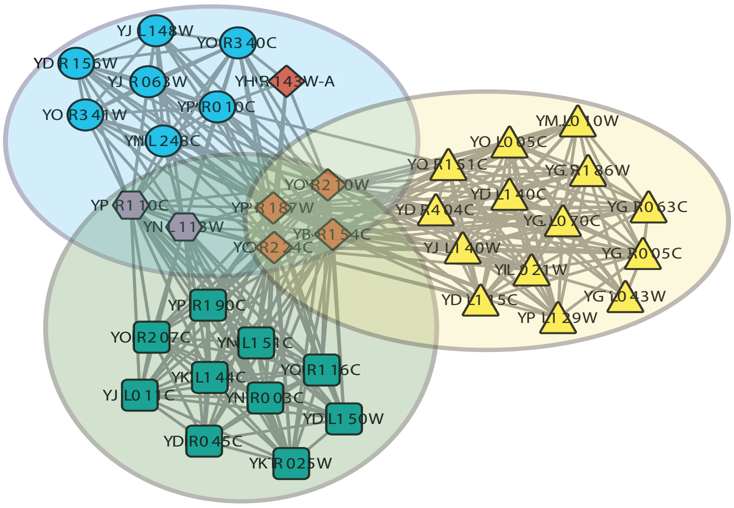

(a)

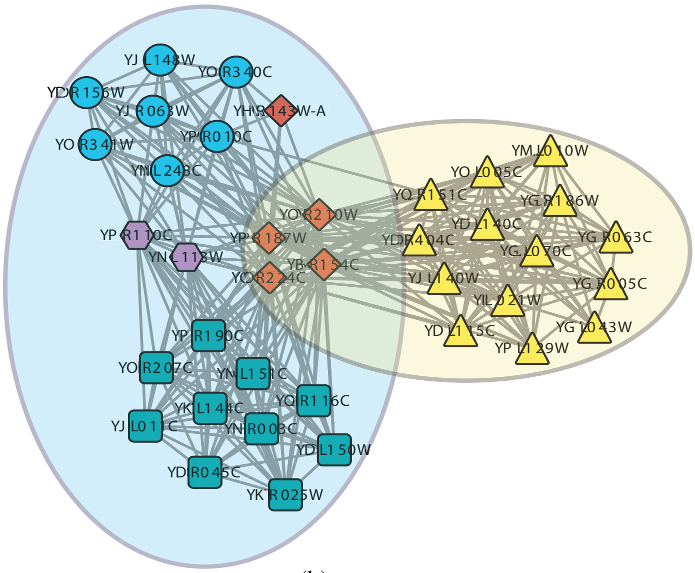

(b)

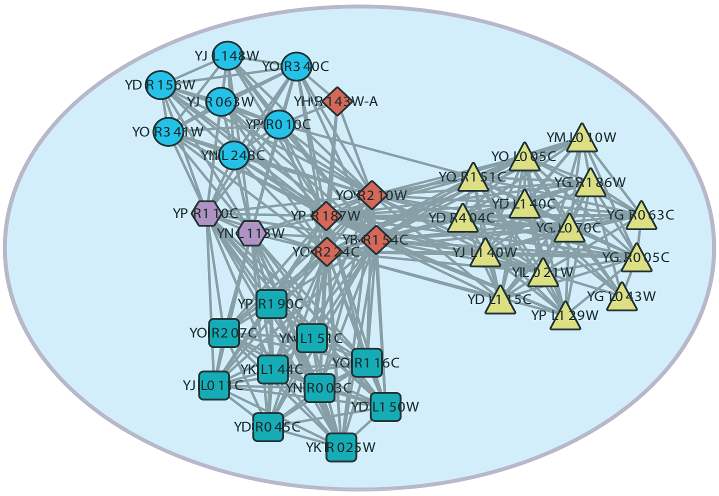

(c)

Figure S8: The DNA-directed RNA polymerase I, II, III complexes detected by GMFTP with different value of parameter λ (a) $\lambda = 4$. (b) $\lambda = 8$. (c) $\lambda = 32$ from the Collins network. Proteins are labeled according to the complex to which they belong: blue circle nodes represent RNA polymerase I; yellow triangle nodes represent RNA polymerase II; green rectangle nodes represent RNA polymerase III. Proteins shared by all the three complexes are labeled with red diamond, and proteins shared by RNA polymerase I and III are labeled with purple hexagon. Shaded areas represent the clusters detected by our model.

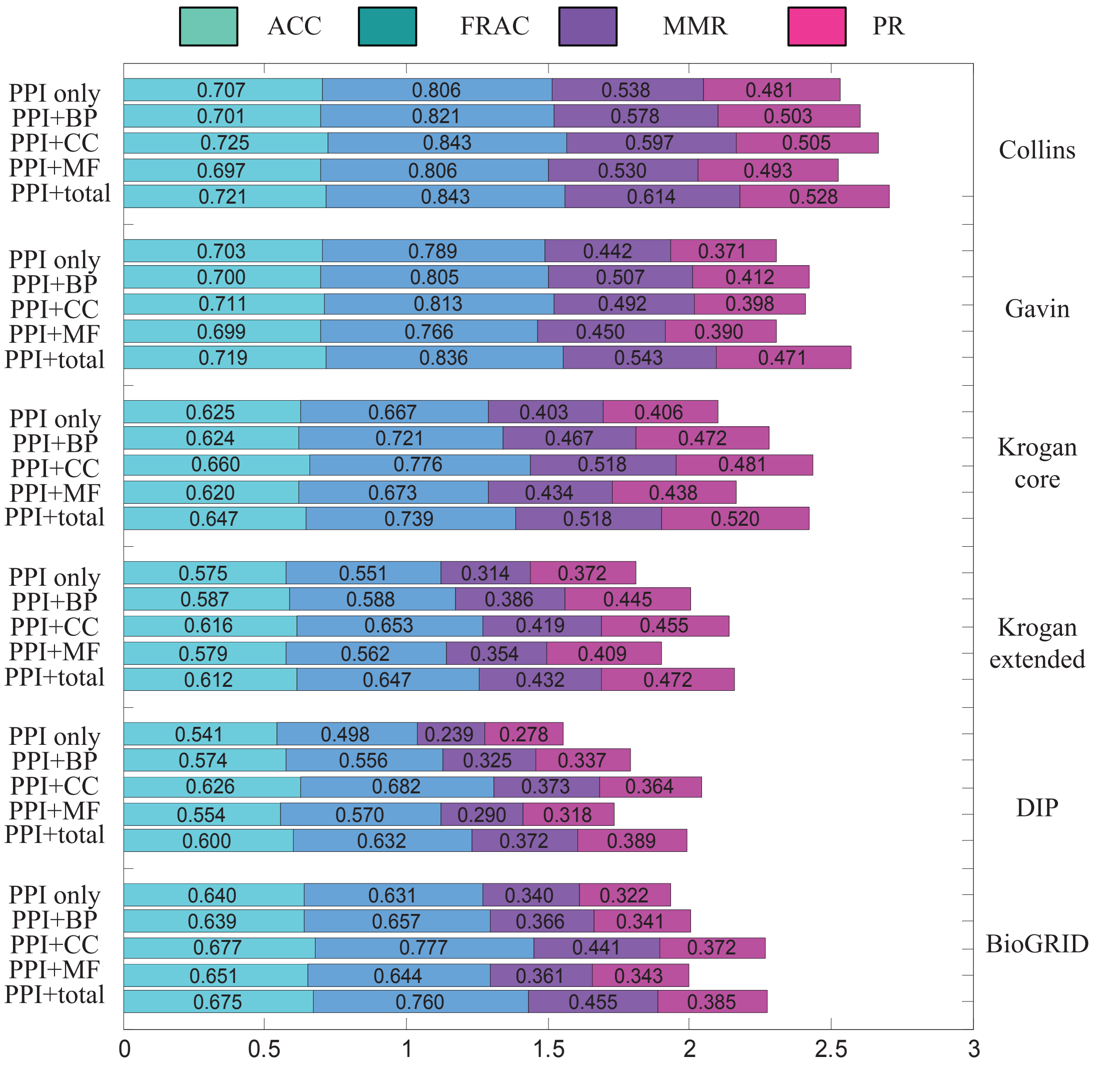

Figure S9: Comparative performance of GMFTP using different categories of functional properties with respect to the SGD gold standard. The total height of each bar is the value of the composite scores of four metrics (ACC, FRAC, MMR and PR) for a given functional property on a given network. Larger scores are better.

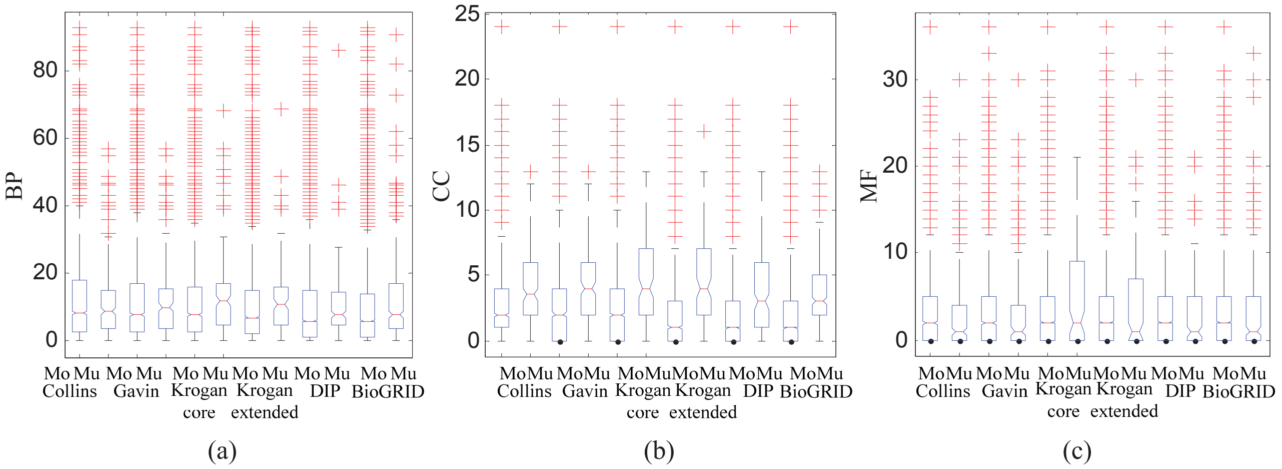

Figure S10: Functional features of mono (Mo)- and multi (Mu)-group proteins detected by GMFTP using the PPI network and the total GO annotation. For each subontology, the distributions of the number of annotated functions of mono (Mo)- and multi (Mu)-grouped proteins are represented by boxplots (line = median). (a) BP. (b) CC. (c) MF.

2 Supplementary Text

2.1 Model parameter estimation

The objective function of GMFTP is

$$\begin{cases} \min_{\Theta,\Psi} & -\sum_{i=1}^{N}\sum_{c=1}^{C} S_i F_{ic}\log\left(1-\exp\left(-\sum_{k=1}^{K}\theta_{ik}\psi_{kc}\right)\right) \\ & +\sum_{i=1}^{N}\sum_{c=1}^{C} S_i\left(1-F_{ic}\right)\left(\sum_{k=1}^{K}\theta_{ik}\psi_{kc}\right) \\ & -\frac{1}{2}\sum_{i,j=1}^{N} A_{ij}\log\left(1-\exp\left(-\sum_{k=1}^{K}\theta_{ik}\theta_{jk}\right)\right) \\ & +\frac{1}{2}\sum_{i,j=1}^{N}\left(1-A_{ij}\right)\left(\sum_{k=1}^{K}\theta_{ik}\theta_{jk}\right) \\ & +\sum_{i=1}^{N}\sum_{k=1}^{K}\lambda\theta_{ik}+\sum_{k=1}^{K}\sum_{c=1}^{C}\lambda\psi_{kc} \\ s.t. & \Theta\geq 0, \Psi\geq 0, \end{cases} \tag{1}$$

where $\Theta \geq 0$ and $\Psi \geq 0$ mean each element $\theta_{ik} \geq 0$ and $\psi_{kc} \geq 0$.

We use the multiplicative updating rules [19] to solve this nonnegative constrained optimization problem. Let ϕ_{ik} and ω_{kc} be the Lagrange multipliers for constraints $\theta_{ik} \geq 0$ and $\psi_{kc} \geq 0$, respectively, and $\Phi = [\phi_{ik}]$, $\Omega = [\omega_{kc}]$. The Lagrange function $\mathcal{L}$ is

$$\begin{aligned} \mathcal{L}\left(\Theta,\Psi,\Phi,\Omega\right) = & -\sum_{i=1}^{N}\sum_{c=1}^{C} S_i F_{ic}\log\left(1-\exp\left(-\sum_{k=1}^{K}\theta_{ik}\psi_{kc}\right)\right)+\sum_{i=1}^{N}\sum_{c=1}^{C} S_i\left(1-F_{ic}\right)\left(\sum_{k=1}^{K}\theta_{ik}\psi_{kc}\right) \\ & -\frac{1}{2}\sum_{i,j=1}^{N} A_{ij}\log\left(1-\exp\left(-\sum_{k=1}^{K}\theta_{ik}\theta_{jk}\right)\right)+\frac{1}{2}\sum_{i,j=1}^{N}\left(1-A_{ij}\right)\left(\sum_{k=1}^{K}\theta_{ik}\theta_{jk}\right) \\ & +\lambda\sum_{i=1}^{N}\sum_{k=1}^{K}\theta_{ik}+\lambda\sum_{k=1}^{K}\sum_{c=1}^{C}\psi_{kc}+\sum_{i=1}^{N}\sum_{k=1}^{K}\phi_{ik}\theta_{ik}+\sum_{k=1}^{K}\sum_{c=1}^{C}\omega_{kc}\psi_{kc}. \end{aligned} \tag{2}$$

The gradients of Lagrange function $\mathcal{L}$ with respect to θ_{ik} and ψ_{kc} are

$$\begin{aligned} \nabla_{\theta_{ik}}\mathcal{L} = & -\sum_{c=1}^{C} S_i F_{ic}\frac{\exp\left(-\sum_{k=1}^{K}\theta_{ik}\psi_{kc}\right)}{1-\exp\left(-\sum_{k=1}^{K}\theta_{ik}\psi_{kc}\right)}\psi_{kc}+\sum_{c=1}^{C} S_i(1-F_{ic})\psi_{kc} \\ & -\sum_{j=1}^{N} A_{ij}\frac{\exp\left(-\sum_{k=1}^{K}\theta_{ik}\theta_{jk}\right)}{1-\exp\left(-\sum_{k=1}^{K}\theta_{ik}\theta_{jk}\right)}\theta_{jk}+\sum_{j=1}^{N}(1-A_{ij})\theta_{jk}+\lambda+\phi_{ik} \\ = & -S_i\sum_{c=1}^{C}\frac{F_{ic}}{1-\exp\left(-\sum_{k=1}^{K}\theta_{ik}\psi_{kc}\right)}\psi_{kc}+S_i\sum_{c=1}^{C}\psi_{kc} \\ & -\sum_{j=1}^{N}\frac{A_{ij}}{1-\exp\left(-\sum_{k=1}^{K}\theta_{ik}\theta_{jk}\right)}\theta_{jk}+\sum_{j=1}^{N}\theta_{jk}+\lambda+\phi_{ik}, \end{aligned} \tag{3}$$

and

$$\begin{aligned} \nabla_{\psi_{kc}}\mathcal{L} = & -\sum_{i=1}^{N} S_i F_{ic}\frac{\exp\left(-\sum_{k=1}^{K}\theta_{ik}\psi_{kc}\right)}{1-\exp\left(-\sum_{k=1}^{K}\theta_{ik}\psi_{kc}\right)}\theta_{ik}+\sum_{i=1}^{N} S_i(1-F_{ic})\theta_{ik}+\lambda+\omega_{kc} \\ = & -\sum_{i=1}^{N} S_i\frac{F_{ic}}{1-\exp\left(-\sum_{k=1}^{K}\theta_{ik}\psi_{kc}\right)}\theta_{ik}+\sum_{i=1}^{N} S_i\theta_{ik}+\lambda+\omega_{kc}. \end{aligned} \tag{4}$$

Since the estimators of θ_{ik} and ψ_{kc} need to satisfy $\nabla_{\theta_{ik}}\mathcal{L} = 0$ and $\nabla_{\psi_{kc}}\mathcal{L} = 0$, we can get

$$\phi_{ik} = S_i\sum_{c=1}^{C}\frac{F_{ic}}{1-\exp\left(-\sum_{k=1}^{K}\theta_{ik}\psi_{kc}\right)}\psi_{kc}-S_i\sum_{c=1}^{C}\psi_{kc}+\sum_{j=1}^{N}\frac{A_{ij}}{1-\exp\left(-\sum_{k=1}^{K}\theta_{ik}\theta_{jk}\right)}\theta_{jk}-\sum_{j=1}^{N}\theta_{jk}-\lambda, \tag{5}$$

and

$$\omega_{kc} = \sum_{i=1}^{N} S_i\frac{F_{ic}}{1-\exp\left(-\sum_{k=1}^{K}\theta_{ik}\psi_{kc}\right)}\theta_{ik}-\sum_{i=1}^{N} S_i\theta_{ik}-\lambda. \tag{6}$$

By the Karush-Kuhn-Tucker (KKT) conditions [18], $\phi_{ik}\theta_{ik} = 0$ and $\omega_{kc}\psi_{kc} = 0$, we get the following equations for θ_{ik} and ψ_{kc}:

$$\theta_{ik}\left(S_i\sum_{c=1}^{C}\psi_{kc} + \sum_{j=1}^{N}\theta_{jk} + \lambda\right) = \theta_{ik}\left(S_i\sum_{c=1}^{C}\frac{F_{ic}}{1-\exp\left(-\sum_{k=1}^{K}\theta_{ik}\psi_{kc}\right)}\psi_{kc} + \sum_{j=1}^{N}\frac{A_{ij}}{1-\exp\left(-\sum_{k=1}^{K}\theta_{ik}\theta_{jk}\right)}\theta_{jk}\right), \tag{7}$$

and

$$\psi_{kc}\left(\sum_{i=1}^{N}S_i\theta_{ik} + \lambda\right) = \psi_{kc}\left(\sum_{i=1}^{N}S_i\frac{F_{ic}}{1-\exp\left(-\sum_{k=1}^{K}\theta_{ik}\psi_{kc}\right)}\theta_{ik}\right). \tag{8}$$

Then, it is easy to obtain the updating formulae for Θ and Ψ, respectively,

$$\theta_{ik} \leftarrow \theta_{ik}\frac{S_i\sum_{c=1}^{C}\frac{F_{ic}}{1-\exp\left(-\sum_{k=1}^{K}\theta_{ik}\psi_{kc}\right)}\psi_{kc} + \sum_{j=1}^{N}\frac{A_{ij}}{1-\exp\left(-\sum_{k=1}^{K}\theta_{ik}\theta_{jk}\right)}\theta_{jk}}{S_i\sum_{c=1}^{C}\psi_{kc} + \sum_{j=1}^{N}\theta_{jk} + \lambda}, \tag{9}$$

and

$$\psi_{kc} \leftarrow \psi_{kc}\frac{\sum_{i=1}^{N}S_i\frac{F_{ic}}{1-\exp\left(-\sum_{k=1}^{K}\theta_{ik}\psi_{kc}\right)}\theta_{ik}}{\sum_{i=1}^{N}S_i\theta_{ik} + \lambda}. \tag{10}$$

To order to help to discuss the computational cost of our model, we rewrite the two updating formulae in a matrix form

$$\Theta \leftarrow \Theta \cdot \left(\frac{diag(S) * \frac{F}{1-exp(-\Theta\Psi)}\psi^T + \frac{A}{1-exp(-\Theta\Theta^T)}\Theta}{diag(S) * repmat(sum(\Psi,2)^T, N, 1) + repmat(sum(\Theta), N, 1) + \lambda}\right), \tag{11}$$

and

$$\Psi \leftarrow \Psi \cdot \left(\frac{\Theta^T diag(S)\frac{F}{1-exp(-\Theta\Psi)}}{repmat(sum(diag(S) * theta)^T, 1, C) + \lambda}\right), \tag{12}$$

where $S = [S_1, S_2, \ldots, S_N]^T$.

2.2 Data sets

We concentrate our study on yeast since it is a well studied model organism for mammalian. Two experimental yeast PPI data sets [14, 17], a combined computational interaction map [10], the interactions derived from DIP [25] and the ones derived from BioGRID [8] are used to test the performance. We refer to these as Gavin, Krogan, Collins, DIP and BioGRID data sets. The Krogan data set is used as two variants: the core data set (referred to as Krogan core) which contains only highly reliable interactions and the extended data set (referred to as Krogan extended) which contains more interactions with less overall reliability. The Collins, Gavin, Krogan core and Krogan extended data sets include edge weights which are estimations of the reliability of interactions. We derive two variants of these four networks: weighted version which includes the weights and unweighted version which ignores the weights. As DIP (version April 6, 2013) and BioGRID (version 3.1.77) provide weights for only a low proportion of the interactions, we treat them as unweighted in a similar manner to that of [21]. We download the Collins, Gavin, Krogan core, Krogan extended and BioGRID networks from the website of Nepusz et al's study (`http://membrane.cs.rhul.ac.uk/static/cl1/cl1_datasets.zip`) [21]. For the DIP network, self-interactions, redundant interactions and interactions involving proteins of which the systematic names are not available are filtered out. For simplicity, we just analyze its the largest connected component. Table S1 lists several topological features of the six networks and shows that they have different structural characterizations. The topological differences between them might can be used to test the generalization and explain the performance differences of a considered approach on different data sets.

We use Gene Ontology [3] as the data source of functional profiles. The Gene Ontology file including three subontologies (biological process (BP), cellular component (CC), and molecular function (MF)) and the GO annotations in SGD [9] are downloaded on 6 April 2013 from `http://www.geneontology.org`. Annotations with the IEA, ND, NAS evidences and the NOT qualifier are excluded. To keep the true-path rule, we process the annotations by associating each protein with its GO terms and all ascendant terms of the associated ones using the ‘ is_a’ and ‘ part of’ relations. To avoid too special and too general functions, we only take into account GO terms with at least 3 and at most 200 associated proteins in the yeast organism. We then derive four categories of functional properties from the annotations of the three individual subontologies and a comprehensive annotation profile which concatenates the ones of all the three subontologies. We refer to them as BP, CC, MF and Total functional profiles, respectively. Proteins that are not associated with any functions considered are regarded as functionally uncharacterized ones. Some statistics of the four functional profiles are presented in Table S2.

We use the CYC2008 [23] and SGD [9] benchmarks as the gold standards of yeast protein complexes. The CYC2008 catalogue is downloaded from `http://wodaklab.org/cyc2008/downloads` on April 6, 2013. For the SGD gold standard, we use the one which is used in [21] and download it from `http://membrane.cs.rhul.ac.uk/static/cl1/cl1$_$gold_standard.zip`. For details of the construction of the benchmark, please refer to [21]. We map

Table S1: Statistics of topological features of the used networks.

	Collins	Gavin	Krogan core	Krogan extended	DIP	BioGRID
Number of proteins	1,622	1,855	2,708	3,672	4,850	5,640
Number of interactions	9,074	7,669	7,123	14,317	21,592	59,748
Weighted	yes	yes	yes	yes	no	no
Average number of neighbors	11.19	8.27	5.26	7.80	8.90	21.19
Centralization	0.0715	0.0215	0.0502	0.0560	0.0553	0.4521
Clustering coefficient	0.5549	0.4675	0.1877	0.1203	0.0985	0.2463
Number of connected components	193	43	63	14	1	1
Density	0.0069	0.0045	0.0019	0.0021	0.0018	0.0038
Diameter	15	13	12	10	10	6

These statistics are calculated using software Cytoscape [29].

Table S2: Statistics of the functional profiles we use.

		Collins	Gavin	Krogan core	Krogan extended	DIP	BioGRID
BP	Number of annotated proteins	1,527	1,712	2,335	3,052	3,826	4,507
	Number of associations	20,232	22,649	31,347	39,940	48,525	54,632
CC	Number of annotated proteins	1,353	1,426	1,761	2,202	2,788	3,284
	Number of associations	5,119	5,444	6,546	8,210	10,472	12,039
MF	Number of annotated proteins	1,047	1,241	1,779	2,360	2,980	3,408
	Number of associations	5,775	6,812	9,500	12,461	15,517	17,489
Total	Number of annotated proteins	1,585	1,790	2,480	3,257	4,111	4,872
	Number of associations	31,126	34,905	47,393	60,611	74,514	84,160

Here "Total" represents the total functional annotations of all the three subontologies.

all the two reference sets onto each PPI network and filter them based on size in a similar manner to that of [21] (`http://membrane.cs.rhul.ac.uk/static/cl1/additional_information.html`). The two gold standards are used independently for evaluation of the methods. The general properties of the reference sets are listed in Table S3.

Table S3: Statistics of the gold standard complexes we use.

		All	Collins	Gavin	Krogan core	Krogan extended	DIP	BioGRID
CYC2008	Number of complexes	408	144	138	164	181	224	236
	Number of proteins	1,627	895	836	850	934	1,128	1,342
	Number of proteins in ≥ 2 complexes	211	140	131	135	142	169	176
SGD	Number of complexes	323	140	132	169	191	229	237
	Number of proteins	1,279	685	629	798	909	1,099	1,168
	Number of proteins in ≥ 2 complexes	332	189	182	240	268	296	306

Here "All" denotes the statistics of each reference set which is not mapped onto the PPI network and filtered in terms of size.

2.3 Evaluation methods

To assess the performance of a considered approach, we need a quantitative criterion to evaluate how a set of predicted complexes matches with a set of reference complexes. Due to the fact that the gold standard complexes (and the predicted complexes if the used algorithm handle overlaps) overlap with each other, a gold standard complex can have a (partial) match with more than one predicted complex and vice versa [21]. It is therefore difficult to find a universal evaluation metric that can work well on this task. In this paper, we use four independent quality measures to evaluate the predicted complexes by comparing them with the reference complexes: accuracy (ACC) [20], fraction of matched complexes (FRAC), maximum matching ratio (MMR) [21] and precision-recall score (PR) [30]. The four metrics assess the performance from different perspectives and have complementary strength.

ACC is a widely used metric which is the the geometric mean of two other measures: the clustering-wise sensitivity (Sn) and the clustering-wise positive predictive value (PPV) [6]. As discussed in [20, 21], the PPV does not evaluate overlapping clusters properly and penalizes the overlapping clustering algorithms. Furthermore, the ACC measure assumes that a complete set of true protein complexes is available, where in reality the gold standards are often incomplete, it therefore puts an approach that predicts many real complexes which do not match with the references at a disadvantage.

FRAC represents the fraction of complexes in the benchmark that are matched by at least one predicted complex with a match score larger than a given threshold w (w is usually set to 0.25) [21]. The FRAC metric pays attention to how the set of reference complexes is matched by a set of predicted complex but ignores how well a reference complex is recovered by a predicted complexes individually.

MMR, which is strongly recommended by the authors, offers a natural way to compare a set of predicted complexes and a set of reference complexes [21]. As discussed by the authors, it penalizes an approach which tends to split a reference complex into more than one part in the predicted complexes. Owing to focusing mainly on test how well the gold standard is matched by the predicted complexes, it does not take the false positive predictions into account. As a result, other methods which quantify the functional homogeneity of predicted complexes are advised to complement the maximum matching ratio [21].

PR score is a metric which pays attention not only to how well the reference complexes are recovered by the predicted complexes (recall) but to how the predicted complexes match to the reference complexes (precision) [30]. Since it takes the size of complexes into account, it penalizes an approach that performs well for small size complex but worse for large size complexes. Similar to ACC, the PR score may be partial to an approach which has a higher precision but a lower recall for an optimal harmonic mean score.

The four metrics are independent and can work together to evaluate the performance of a complexes detection approach. For ACC, FRAC and MMR, the python script for the calculation of quality scores is downloaded from `http://membrane.cs.rhul.ac.uk/static/cl1/cl1_reproducibility.zip`. We implement the Matlab code for the calculation of PR score according to the formulations described in [30].

We also test the functional homogeneity of predicted complexes, following the method of Nepusz et al [21]. The hypergeometric distribution is used to calculate the P-value of biological relevance for a predicted complex and a given functional term. The overrepresentation score of an approach is calculated by the ratio between the number of predicted complexes with at least one enriched annotation and the total number of predicted complexes. The Bonferroni method is used to adjust the P-value to keep that the overall significance level of the test at 0.05. We implement the calculation using the web service of GO Term Finder (`http://go.princeton.edu/cgi-bin/GOTermFinder`) [5]. Here we also use the yeast GO annotations downloaded on 6 April 2013 as the data source of functional classifications and annotations, which are previously used to construct the functional profiles. Similarly, annotations with the IEA, ND, NAS evidences are not taken into account. Since one may argue that using GO annotations with evidence IPI presents a case of circular reasoning as these annotations are also inferred from physical interactions, the annotations with IPI evidence code are also excluded. In order to avoid evaluation bias, we only use the GO annotations to assess the functional homogeneity of complexes predicted using only the PPI networks rather than those using both the PPI networks and the functional profiles.

2.4 Convergence and computational complexity analysis

We have developed an iterative algorithm to solve the optimization problem of GMFTP based on multiplicative updating rules. It is known that the multiplicative updating rules are special cases of gradient descent methods with an automatic step parameter selection for guaranteeing the nonnegativity of parameters [7]. It may therefore be able to prove that the objective function of our model is nonincreasing under the update and that the iterative algorithm is guaranteed to find at least locally optimal solutions by constructing an auxiliary function similar to that used in [27, 7]. Instead of proving this in theory, we validate the convergence experimentally.

For the six networks considered in this paper, we consider the two extreme cases of functional properties: PPI only which represents using only the PPI networks and PPI+total which represents using both the PPI networks and the total functional profiles. From Fig. S11, we find that the objective function decreases sharply first and then becomes smoothly under the update. For small size data sets (e.g., Collins, Gavin and Krogan core), the updating process converges within 400 iterations (the relative change of the objective function is less than 10^{-6}); for large size data sets (e.g., DIP and BioGRID), the procedure can not converge within 400 iterations but the change of the objective function is also negligible. These results demonstrate the convergence of our parameter estimation algorithm.

The computational cost of GMFTP is mainly determined by Equations (11) and (12). In Equation (11), computation of $diag(S) * \frac{F}{1-exp(-\Theta\Psi)}\Psi^T$ requires $O(NKC)$ times; computation of $\frac{A}{1-exp(-\Theta\Theta^T)}\Theta$ requires $O(N^2K)$ times; computation of $diag(S)*repmat(sum(\Psi,2)^T, N, 1)$ requires $O(K(N+C))$ times; computation of $repmat(sum(\Theta), N, 1)$ requires $O(NK)$ times. Thus, each update of Θ takes $O\left(KN(N+C)\right)$ times. In Equation (12), computation of $\Theta^T diag(S)\frac{F}{1-exp(-\Theta\Psi)}$ requires $O(NKC)$ times; computation of $repmat(sum(diag(S)*Theta)^T, 1, C)$ requires $O(K(N+C))$ times. Thus, each update of Ψ takes $O(NKC)$ times. Therefore the total time cost of GM-FTP is $O\left(KNT(N+C)\right)$, where T is the number of iterations.

In real world situations, the PPI networks and functional profiles usually are extremely sparse. The overall cost therefore can be reduced. In fact, computation cost of $\frac{F}{1-exp(-\Theta\Psi)}$ can be reduced to $O(KR)$ times, where R is the number of functional associations between proteins and functions. This is because it does not need to compute $(1-exp(-\Theta\Psi))_{ic}$ if $F_{ic}=0$. Computation of $\frac{F}{1-exp(-\Theta\Psi)}\Psi^T$ requires only $O(KR)$ times due to the fact that $\frac{F}{1-exp(-\Theta\Psi)}$ only contains R non-zero elements. Thus, computation of $diag(S) * \frac{F}{1-exp(-\Theta\Psi)}\Psi^T$ requires only $O(KR)$ times. Similarly, computation cost of $\frac{A}{1-exp(-\Theta\Theta^T)}\Theta$ can be reduced to $O(KE)$, where E is the number of interactions between proteins. As a result, time cost of updating Θ can be reduced to $O(K(N+C+E+R))$. Similarly, time cost of updating Ψ can be reduced to $O(K(N+C+R))$. The total cost can therefore be reduced to $O(KT(N+C+E+R))$.

Table S4 presents the time cost of our parameter estimation method. For each PPI network, we only consider the results using 'PPI only' and 'PPI+total' functional profiles. We repeat the updating process 100 times, and compute the average time cost for the entire process of parameter estimation and for per update. We implement the algorithm using Matlab in

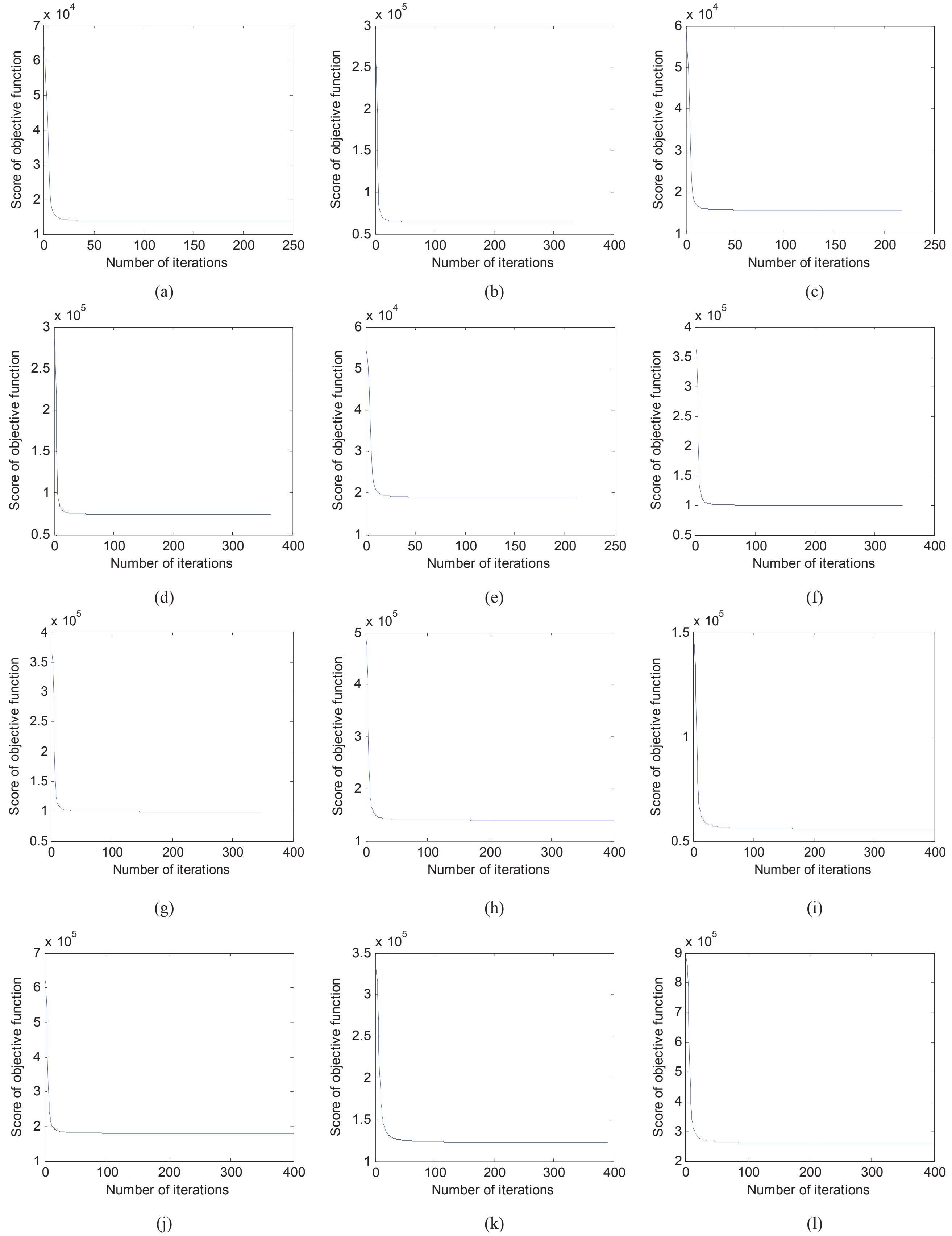

Figure S11: Convergence analysis of parameter estimation. For each figure, the x-axis denotes the number of iterations, and the y-axis denotes the value of the objective function. (a) Collins with PPI only. (b) Collins with PPI+total. (c) Gavin with PPI only. (d) Gavin with PPI+total. (e) Krogan core with PPI only. (f) Krogan core with PPI+total. (g) Krogan extended with PPI only. (h) Krogan extended with PPI+total. (i) DIP with PPI only. (j) DIP with PPI+total. (k) BioGRID with PPI only. (l) BioGRID with PPI+total.

a workstation with Intel 4 CPU (3.40 GH $\times$ 4) and 16 GB RAM. Each update costs at most 3.25 seconds and the entire estimation takes less than 1300 seconds when we set the maximum number of iterations to 400.

Table S4: Average time cost of GMFTP for estimating model parameters.

Property	Type	Collins	Gavin	Krogan core	Krogan extended	DIP	BioGRID
PPI only	Per update	0.32	0.36	0.60	0.90	1.38	1.82
	Entire estimation	78.20	79.16	127.42	361.87	550.02	708.54
PPI+total	Per update	0.76	0.88	1.32	1.85	2.62	3.25
	Entire estimation	252.48	320.77	456.68	740.31	1049.05	1299.39

2.5 Effect of random restarts

Due to the fact that the objective function of GMFTP is not convex, the multiplicative updating rules-based iterative algorithm does not necessarily converge to the global minimum. In the main text, to guard against the possibility of getting stuck in a local minimum to some degree, we repeat the entire calculation 100 times with random restarts and choose the result that gives the lowest value of the objective function. We limit the number of repetitions to 100 because of the time cost of more repetitions. As a result, we can not guarantee the final estimator is the globally optimum solution and the result is not deterministic. We therefore focus on the variability in results of multiple run of this procedure. The entire repetition procedure is implemented 20 times of which each time uses 10 random restarts to obtain a result.

Here we also just consider the 'PPI only' and 'PPI+total' functional profiles. The mean, median, maximum, minimum and standard deviation of the evaluation results of these 20 experiments are use to assess the stability. Due to the higher value of the standard deviation, the performances measured using the FRAC and MMR criteria are more sensitive to initialization conditions than those measured using the ACC and PR criteria (Table S5). The low value of standard deviation shows that the performance of GMFTP does not have a big change with different initializations. However, the differences between the maximum and the minimum value of evaluation scores show that we would obtain better performance if we implement GMFTP with more restarts.

2.6 Effect of K

In the main text, we do not discuss the effect of K, which is assumed to be the maximum possible number of complexes. Since we have placed an exponential prior over Θ and Ψ, which leads the fact that the estimators of model parameters are sparse automatically. We therefore set $K = 1000$ as the possible number of complexes in all the six data sets. Here, we try to test whether the choice of K has a significant influence on the performance of GMFTP. We fix λ to 4 and run GMFTP with various values of K ($K \in \{500, 1000, 1500\}$). The performance is evaluated by comparing the predicted complexes to the gold standard complexes.

For the six networks, we only consider the two extreme cases of functional properties (PPI only and PPI+total). The results listed in Table S6 show that the performances vary a little with respect to different value of K. For each PPI network and category of functional profile, there is not a deliberately selected K that can dominate the other two cases in terms of all the four evaluation metrics and the three gold standards. We may therefore conjecture that the variability in performance may come from the local minimum of the solution as discussed in the above section. These results may suggest that the performance of GMFTP would not have a big change if we set K to a value which is higher than the true number of complexes.

2.7 Parameter settings of compared algorithms

In this paper, in order to evaluate the performance of GMFTP in detecting protein complexes, we compare it with ten competitive methods: Affinity propagation (AP) [13], CFinder [1], ClusterONE [21], COAN [31], Linkcomm [2], MCL [11], MCODE [4], MINE [24], SPICi [15] and SR-MCL [28]. Table S7 lists the websites where we download the softwares of these algorithms, the version numbers of these softwares and several indications about whether these algorithms could be applied to weighted PPI networks or handle overlaps. Before describing the parameter settings for each algorithm, we declare several general consideration first. Since the performance of each algorithm depends on the choice of its inherent parameters and the data set under consideration, for all the considered algorithms, we optimize the parameters that yield the best results in a similar way to that of [21]. To avoid evaluation bias, we also consider the following three criteria:

- Four quality metrics (ACC, FRAC, MMR and PR score) are used to evaluate the performance of each algorithm.
- Two different gold standards (the CYC2008 complexes and the SGD complexes) are used.
- For each algorithm, the final results are obtained by choosing the parameters that yield the best performance which are measured by the MMR metric on the gold standard that is being tested in the benchmark (CYC2008 or SGD).

We briefly review the main features of these algorithms and the setting of parameters for each algorithm in the following text.

Table S5: Stability analysis of GMFTP with respect to different random restarts.

			CYC2008				SGD			
Data set	Property	Statistics	ACC	FRAC	MMR	PR	ACC	FRAC	MMR	PR
Collins	PPI only	Mean	0.762	0.851	0.563	0.575	0.706	0.790	0.516	0.473
		Median	0.763	0.847	0.565	0.574	0.706	0.787	0.517	0.473
		Max	0.767	0.882	0.591	0.593	0.709	0.806	0.538	0.481
		Min	0.755	0.833	0.539	0.561	0.699	0.769	0.494	0.464
		Std	0.004	0.014	0.015	0.010	0.003	0.011	0.013	0.005
	PPI+total	Mean	0.778	0.876	0.634	0.646	0.715	0.829	0.586	0.525
		Median	0.778	0.882	0.635	0.647	0.715	0.828	0.584	0.528
		Max	0.788	0.896	0.659	0.660	0.722	0.866	0.614	0.536
		Min	0.769	0.847	0.610	0.639	0.709	0.791	0.554	0.511
		Std	0.005	0.017	0.014	0.006	0.005	0.022	0.017	0.008
Gavin	PPI only	Mean	0.741	0.820	0.486	0.454	0.696	0.759	0.430	0.367
		Median	0.741	0.822	0.489	0.455	0.696	0.758	0.434	0.369
		Max	0.746	0.848	0.500	0.462	0.703	0.789	0.443	0.376
		Min	0.737	0.790	0.461	0.448	0.691	0.734	0.394	0.357
		Std	0.003	0.019	0.011	0.005	0.004	0.015	0.014	0.006
	PPI+total	Mean	0.758	0.868	0.588	0.567	0.710	0.821	0.538	0.469
		Median	0.759	0.870	0.587	0.566	0.713	0.824	0.532	0.471
		Max	0.769	0.891	0.615	0.583	0.719	0.844	0.572	0.487
		Min	0.750	0.841	0.569	0.545	0.696	0.797	0.517	0.456
		Std	0.006	0.014	0.017	0.012	0.007	0.014	0.019	0.010
Krogan core	PPI only	Mean	0.717	0.735	0.457	0.480	0.613	0.644	0.384	0.395
		Median	0.716	0.735	0.456	0.481	0.613	0.645	0.383	0.394
		Max	0.723	0.756	0.474	0.491	0.625	0.667	0.403	0.406
		Min	0.710	0.707	0.442	0.469	0.607	0.618	0.371	0.386
		Std	0.004	0.014	0.010	0.008	0.005	0.015	0.009	0.005
	PPI+total	Mean	0.756	0.774	0.545	0.636	0.640	0.709	0.491	0.515
		Median	0.755	0.771	0.543	0.635	0.640	0.709	0.494	0.512
		Max	0.768	0.805	0.573	0.649	0.647	0.739	0.520	0.531
		Min	0.746	0.750	0.523	0.622	0.633	0.685	0.464	0.501
		Std	0.006	0.017	0.014	0.008	0.005	0.014	0.015	0.007
Krogan extended	PPI only	Mean	0.683	0.606	0.376	0.467	0.569	0.523	0.305	0.372
		Median	0.683	0.613	0.373	0.468	0.568	0.524	0.306	0.371
		Max	0.692	0.652	0.399	0.483	0.576	0.551	0.314	0.382
		Min	0.673	0.558	0.354	0.446	0.564	0.497	0.295	0.366
		Std	0.006	0.027	0.015	0.010	0.005	0.016	0.007	0.005
	PPI+total	Mean	0.727	0.684	0.481	0.595	0.601	0.619	0.421	0.469
		Median	0.729	0.688	0.483	0.597	0.601	0.623	0.421	0.469
		Max	0.741	0.696	0.497	0.608	0.612	0.647	0.432	0.480
		Min	0.716	0.669	0.464	0.574	0.590	0.594	0.405	0.460
		Std	0.007	0.012	0.009	0.011	0.007	0.017	0.008	0.007
DIP	PPI only	Mean	0.629	0.533	0.289	0.322	0.535	0.457	0.232	0.271
		Median	0.632	0.540	0.293	0.324	0.536	0.455	0.231	0.273
		Max	0.642	0.580	0.302	0.329	0.543	0.498	0.248	0.277
		Min	0.613	0.491	0.269	0.311	0.522	0.444	0.224	0.259
		Std	0.009	0.019	0.011	0.006	0.006	0.008	0.007	0.006
	PPI+total	Mean	0.700	0.675	0.422	0.462	0.597	0.621	0.369	0.378
		Median	0.701	0.670	0.419	0.462	0.597	0.621	0.369	0.377
		Max	0.708	0.705	0.431	0.474	0.605	0.646	0.382	0.390
		Min	0.690	0.652	0.414	0.448	0.589	0.596	0.358	0.365
		Std	0.006	0.019	0.006	0.007	0.005	0.016	0.008	0.008
BioGRID	PPI only	Mean	0.716	0.674	0.365	0.370	0.634	0.609	0.318	0.310
		Median	0.716	0.672	0.365	0.369	0.634	0.605	0.317	0.310
		Max	0.723	0.699	0.381	0.380	0.640	0.631	0.340	0.322
		Min	0.708	0.653	0.348	0.358	0.626	0.588	0.300	0.305
		Std	0.004	0.015	0.011	0.008	0.004	0.015	0.013	0.005
	PPI+total	Mean	0.748	0.753	0.458	0.438	0.663	0.726	0.428	0.373
		Median	0.747	0.752	0.459	0.437	0.664	0.727	0.429	0.373
		Max	0.754	0.788	0.489	0.450	0.675	0.760	0.455	0.385
		Min	0.741	0.733	0.416	0.427	0.651	0.678	0.393	0.365
		Std	0.004	0.017	0.019	0.008	0.007	0.026	0.019	0.006

Affinity propagation

In the affinity propagation algorithm (AP) [13], each node is assigned a parameter called preference, which controls the likelihood of that node being an exemplar (i.e., a representative element of a cluster). It is a common practice to set the preference value equal for all nodes. In this paper, the optimal preference value is determined by trying different values

Table S6: Performance of GMFTP with respect to different values of K.

			CYC2008				SGD			
Data set	Property	K	ACC	FRAC	MMR	PR	ACC	FRAC	MMR	PR
Collins	PPI only	500	0.764	0.854	0.560	0.587	0.710	0.784	0.521	0.488
		1000	0.765	0.868	0.591	0.593	0.707	0.806	0.538	0.481
		1500	0.766	0.854	0.575	0.579	0.710	0.799	0.524	0.478
	PPI+total	500	0.779	0.882	0.641	0.665	0.715	0.836	0.603	0.543
		1000	0.788	0.890	0.659	0.651	0.721	0.843	0.614	0.528
		1500	0.774	0.896	0.661	0.648	0.708	0.843	0.601	0.530
Gavin	PPI only	500	0.742	0.790	0.472	0.460	0.694	0.750	0.411	0.367
		1000	0.742	0.841	0.489	0.457	0.703	0.789	0.442	0.371
		1500	0.747	0.819	0.497	0.456	0.696	0.742	0.433	0.367
	PPI+total	500	0.761	0.855	0.588	0.576	0.716	0.828	0.523	0.469
		1000	0.768	0.877	0.594	0.570	0.719	0.836	0.543	0.471
		1500	0.769	0.855	0.593	0.580	0.718	0.813	0.522	0.459
Krogan core	PPI only	500	0.715	0.713	0.442	0.487	0.613	0.655	0.377	0.402
		1000	0.722	0.756	0.474	0.491	0.625	0.667	0.403	0.406
		1500	0.714	0.726	0.454	0.468	0.616	0.642	0.383	0.386
	PPI+total	500	0.755	0.762	0.540	0.624	0.631	0.727	0.496	0.510
		1000	0.768	0.805	0.573	0.649	0.647	0.739	0.518	0.520
		1500	0.765	0.799	0.570	0.651	0.640	0.679	0.496	0.522
Krogan extended	PPI only	500	0.680	0.597	0.346	0.461	0.565	0.508	0.289	0.370
		1000	0.692	0.652	0.398	0.469	0.575	0.551	0.314	0.372
		1500	0.688	0.608	0.381	0.479	0.570	0.519	0.302	0.376
	PPI+total	500	0.719	0.658	0.449	0.577	0.605	0.578	0.398	0.465
		1000	0.745	0.696	0.487	0.601	0.612	0.647	0.432	0.472
		1500	0.725	0.713	0.499	0.611	0.610	0.636	0.434	0.484
DIP	PPI only	500	0.618	0.491	0.257	0.309	0.533	0.448	0.218	0.266
		1000	0.639	0.580	0.296	0.329	0.541	0.498	0.239	0.278
		1500	0.629	0.527	0.306	0.328	0.536	0.453	0.245	0.277
	PPI+total	500	0.709	0.679	0.420	0.456	0.625	0.632	0.371	0.383
		1000	0.704	0.705	0.430	0.474	0.600	0.632	0.372	0.389
		1500	0.695	0.705	0.452	0.478	0.596	0.637	0.393	0.402
BioGRID	PPI only	500	0.712	0.623	0.329	0.366	0.628	0.579	0.281	0.304
		1000	0.723	0.687	0.377	0.378	0.640	0.631	0.340	0.322
		1500	0.722	0.703	0.398	0.377	0.632	0.635	0.334	0.312
	PPI+total	500	0.738	0.678	0.413	0.409	0.664	0.682	0.379	0.348
		1000	0.754	0.750	0.474	0.448	0.675	0.760	0.455	0.385
		1500	0.741	0.801	0.501	0.448	0.664	0.760	0.465	0.383

(ranges from -1 to 1 with 0.1 increment) and setting on the preference value that results in the best quality score. Affinity propagation can handle both weighted and unweighted networks, therefore, besides the original PPI networks, we design a heuristic comparison to test the performance of Affinity propagation when incorporating GO annotations into complexes detection process. We employ three widely used measures Jiang, Kappa and Lin to weight the PPI networks, and implement Affinity propagation on these weighted networks to detect protein complexes. The optimal value of preference for each data set is shown in Table S8.

CFinder

Adamcsek *et al.* [1] provided a software called CFinder which is based on Clique Percolation Method (CPM) [22] to detect overlapping modules in biological networks. CPM detects overlapping clusters by finding k-clique percolation communities. Therefore, a key parameter of CFinder is the size of k-clique. In this paper, for each PPI network, we test CFinder with k-clique size from 3 to 10, step size by 1. For BioGRID network, since CFinder did not give any result within 48 hours, we set an optional time limit (10 seconds) for the time to spend on each node of the network, such that it can analyze the network efficiently. Table S9 lists the optimal values of parameter k for each PPI network. The original CPM method can only handle unweighted networks. Even though Farkas et al have proposed a weighted extension of CFinder [12], the computational cost of the new variant is more prohibitive and can not analyze Collins network in 48 hours [21]. Therefore we just list the results on the unweighted PPI networks.

ClusterONE

ClusterONE is recently proposed by Nepusz *et al.* [21] to detect overlapping protein complexes in PPI networks based on overlapping neighborhood expansion. ClusterONE can deal with weighted and unweighted networks, therefore, besides the original network, we also test its performance on the weighted networks which are constructed by assigning weights to

Table S7: Characteristics of the compared algorithms.

Algorithm	Downloading website	Version	weights supported	overlap supported
AP	`http://www.psi.toronto.edu/index.php?q=affinity%20propagation`		√	
CFinder	`http://cfinder.org/`	2.0.5		√
ClusterONE	`http://www.paccanarolab.org/cluster-one/index.html`	0.94	√	√
COAN	`http://www.plosone.org/article/info:doi/10.1371/journal.pone.0062077`		√	
Linkcomm	`http://barabasilab.neu.edu/projects/linkcommunities/`	2010-02-25		√
MCL	`http://micans.org/mcl/`	09-308	√	
MCODE	`http://baderlab.org/Software/MCODE`	1.32		√
MINE	`http://www.biomedcentral.com/1471-2105/12/192`	1.5		√
SPICi	`http://compbio.cs.princeton.edu/spici/`		√	
SR-MCL	`http://www.cse.ohio-state.edu/~shihy/`		√	√

Table S8: The value of preference selected for Affinity propagation

Gold Standard	Network	PPI (nw)	PPI (w)	PPI + BP			PPI + CC			PPI + MF			PPI + Total		
				Jiang	Kappa	Lin	Jiang	Kappa	Lin	Jiang	Kappa	Lin	Jiang	Kappa	Lin
CYC2008	Collins	0.9	0.5	0.4	0.1	0.2	0.9	0.5	0.8	0.3	0.1	0.9	0.3	0.1	0.1
	Gavin	0.6	0.2	0.7	0.3	0.7	0.4	0.7	0.4	0.1	0.2	0.2	0.3	0.3	0.3
	Krogan core	0.4	0.7	0.5	0.4	0.6	0.4	0.6	0.9	0.7	0.3	0.6	0.1	0.3	0.4
	Krogan extended	0.9	0.7	0.4	0.4	0.9	0.8	0.6	0.8	0.6	0.2	0.8	0.4	0.3	0.5
	DIP	0.9	-	0.7	0.4	0.8	0.4	0.9	0.9	0.2	0.2	0.4	0.7	0.3	0.8
	BioGRID	0.1	-	0.7	0.3	0.7	0.5	0.7	0.7	0.9	0.3	0.5	0.4	0.3	0.9
SGD	Collins	0.9	0.5	0.8	0.1	0.2	0.9	0.5	0.9	0.3	0.1	0.9	0.3	0.1	0.7
	Gavin	0.1	0.2	0.9	0.1	0.7	0.3	0.7	0.4	0.6	0.4	0.1	0.6	0.2	0.9
	Krogan core	0.4	0.6	0.4	0.3	0.5	0.4	0.6	0.9	0.8	0.1	0.8	0.6	0.3	0.4
	Krogan extended	0.9	0.6	0.4	0.2	0.9	0.9	0.6	0.9	0.6	0.2	0.7	0.4	0.3	0.5
	DIP	0.9	-	0.8	0.4	0.9	0.4	0.8	0.8	0.2	0.1	0.5	0.6	0.5	0.5
	BioGRID	0.9	-	0.7	0.3	0.8	0.2	0.7	0.9	0.9	0.3	0.1	0.4	0.3	0.6

Table S9: Parameters selected for CFinder.

Gold Standard	Collins	Gavin	Krogan core	Krogan extended	DIP	BioGRID
CYC2008	3	4	3	4	5	6
SGD	3	4	3	4	5	6

Table S10: Parameters selected for COAN.

Gold Standard	Collins	Gavin	Krogan core	Krogan extended	DIP	BioGRID
CYC2008	0.7	0.6	0.6	0.6	0.8	0.7
SGD	0.7	0.6	0.6	0.7	0.7	0.7

Table S11: The value of inflation selected for MCL.

Gold Standard	Network	PPI (nw)	PPI (w)	PPI + BP			PPI + CC			PPI + MF			PPI + Total		
				Jiang	Kappa	Lin	Jiang	Kappa	Lin	Jiang	Kappa	Lin	Jiang	Kappa	Lin
CYC2008	Collins	3.2	5.0	5.0	3.4	5.0	4.2	4.6	4.8	1.8	2.6	2.6	4.0	4.4	2.8
	Gavin	3.6	3.4	3.6	4.8	3.2	4.2	2.8	3.2	2.0	3.0	2.2	4.0	3.2	5.0
	Krogan core	2.6	2.4	2.6	3.8	2.6	2.4	2.4	2.2	1.8	2.0	1.8	2.8	2.4	2.4
	Krogan extended	2.2	2.4	2.6	2.4	2.2	2.8	2.6	2.4	1.8	2.4	1.8	3.0	2.6	2.2
	DIP	2.0	-	2.2	2.2	2.2	2.2	2.0	2.2	2.0	2.4	2.0	2.4	2.6	2.2
	BioGRID	3.2	-	3.0	2.4	3.2	2.8	2.6	2.8	2.6	2.4	2.4	2.8	2.6	2.8
SGD	Collins	2.8	5.0	5.0	3.4	5.0	4.2	5.0	4.8	1.8	2.6	2.6	4.0	3.4	2.6
	Gavin	2.8	4.6	3.6	4.6	4.2	4.2	2.8	3.2	3.4	2.8	3.0	4.4	2.8	5.0
	Krogan core	2.8	2.0	2.6	2.0	2.4	2.2	2.4	2.2	1.8	1.8	1.8	2.8	2.4	2.4
	Krogan extended	2.2	2.6	2.6	2.0	2.2	2.0	2.0	1.8	1.8	1.8	1.8	2.0	2.0	2.2
	DIP	2.2	-	2.4	2.2	2.2	2.2	2.6	2.0	2.4	2.4	2.4	2.4	2.6	2.0
	BioGRID	3.2	-	2.4	2.8	2.6	2.8	2.4	2.8	2.6	2.8	2.6	3.0	2.6	2.6

interactions according to the GO annotations. As suggested by the authors, we do not tune the parameters for a particular network. Thus, we use the default settings of parameters in the software.

COAN

Zhang *et al.* [31] utilized the protein-protein interaction data and Gene Ontology to construct ontology augmented networks, and proposed a novel method (clustering based on ontology augmented networks (COAN)) to predict protein complexes. COAN can take into account both the topological structure of the PPI network and the similarity of GO slims annotation. The key parameter of COAN is the $extend\_thres$ which is used to expand seed complexes. In this study, we use GO slims annotation data provided by the authors in their Supporting Information. As suggested by the authors, the range of $extend\_thres$ is from 0 to 1 with 0.1 increment. The optimal value of $extend\_thres$ for each PPI network is shown in Table S10.

Linkcomm

Linkcomm [2] is a landmark method in the field of community detection. Through reinventing communities as groups of links rather than nodes, it successfully captures the organizing principles of overlapping communities and hierarchy. It has a parameter which is used to cut the dendrogram. In this study, we use the default parameter, such that it automatically cuts the hierarchical tree at the point where the density function of the partition is maximized. The original method is implemented on unweighted networks, and Kalinka and Tomancak then extended it to handle networks that are weighted [16]. Here we implement the unweighted version such that the comparative experiments are in accordance with the original method.

MCL

Markov Clustering Algorithm (MCL) [11] is a competing protein complex detection algorithm and has been implemented in different languages, such as JAVA, R and C. The key parameter of MCL is inflation, which tunes the granularity of clustering. Here, we try different values of inflation, ranges from 1.2 to 5.0 with 0.2 increment. The optimal value of inflation for each PPI network is shown in Table S11. MCL can handle weighted networks, thus we also list its results on the weighted PPI networks constructed.

MCODE

MCODE [4] is an effective approach for detecting protein complexes. It consists of three stages: vertex weighting, complex prediction and optionally post-processing. Among its inherent parameters, the depth limit parameter controls the duration of the augment process. The node score cutoff parameter controls the difference that can be tolerance between scores of proteins within the same complex, and it is closely related to the size of the complex. There are two possible post-processing operations: haircut and fluffing. MCODE is able to produce overlapping complexes in the fluffing case, but we experimentally find that when fluffing is turned off, MCODE always has better performance. Therefore, we turn off the fluffing process in this study. We try all possible combinations of the following parameters:

- Depth limit: 3, 4, 5

Table S12: Parameters selected for MCODE.

Gold Standard	Parameter	Collins	Gavin	Krogan core	Krogan extended	DIP	BioGRID
CYC2008	Depth limit	3	3	3	3	3	3
	Node score cutoff	0.2	0.1	0.3	0.2	0.1	0.1
	Haircut	on	on	on	on	on	on
	Fluffing	off	off	off	off	off	off
	Node density cutoff	N/A	N/A	N/A	N/A	N/A	N/A
SGD	Depth limit	3	3	3	3	3	3
	Node score cutoff	0.3	0.2	0.3	0.3	0.1	0.1
	Haircut	on	on	on	on	on	on
	Fluffing	off	off	off	off	off	off
	Node density cutoff	N/A	N/A	N/A	N/A	N/A	N/A

Table S13: Parameters for MINE.

Gold Standard	Parameter	Collins	Gavin	Krogan core	Krogan extended	DIP	BioGRID
CYC2008	Depth limit	3	3	3	3	3	3
	Node score cutoff	0.1	0.1	0.1	0.1	0.1	0.1
	Modularity score cutoff	0.2	0.8	0.1	0.1	0.1	0.1
SGD	Depth limit	3	3	3	3	3	3
	Node score cutoff	0.1	0.1	0.3	0.1	0.1	0.1
	Modularity score cutoff	0.1	0.8	0.2	0.2	0.1	0.1

- Node score cutoff: 0.1 to 1.0 with a step size of 0.1
- Haircut: on or off
- Fluffing: on or off
- Node density cutoff: 0, 0.1, 0.2

Since MCODE can not deal with weighted networks, we list the optimal parameters of MCODE for the unweighted verion of each PPI network in Table S12.

MINE

MINE [24] can identify highly modular sets of proteins within highly interconnected PPI networks. The key parameters of MINE are node score cutoff and modularity score cutoff. We try different value of node score cutoff and modularity score cutoff (from 0.1 to 1 with 0.1 as the step size) and 3 settings of depth limit (3, 4, 5). For the other parameters, without stating, we use the default values in the software. MINE can not handle weighted networks neither, thus we apply it on the unweighted PPI networks. The optimal values of the parameters of MINE for each PPI network are listed in Table S13.

SPICi

SPICi [15] is a computationally efficient local network clustering algorithm for large biological networks, which can be applied on PPI networks for complex detection. SPICi can handle weighted networks, so we apply it on the weighted and unweighted PPI networks to test its performance. SPICi has two parameters: the density threshold and the support threshold. Here, we try different values of density threshold, ranges from 0.1 to 1 with 0.1 increment. For the other parameters, we use the default settings in the software. Table S14 lists the optimal value of density parameter for each PPI networks.

SR-MCL

In order to redress the limitation of MCL [11] and its variants (e.g. regularized MCL) [26] that it only supports hard clustering, Shih and Parthasarathy proposed a soft variation of Regularized MCL (called SR-MCL) [28]. There are four parameters that need to be tuned: the balance parameter b, the inflation parameter r, the penalized ratio β and the number of iteration t. Because it would take a very long time to find the optimal values of the four parameters by grid searching, SR-MCL is set to the default values as suggested by the authors. Even though the method can be implemented on weighted networks, we experimentally find that the software provided by the authors can only be performed on weighted networks for which the edge weights are integers (We also discussed this point with authors). Because the edge weights considered in this study are in $[0, 1]$, we only implement it on the unweighted networks.

Table S14: The value of density selected for SPICi

Gold Standard	Network	PPI (nw)	PPI (w)	PPI + BP			PPI + CC			PPI + MF			PPI + Total		
				Jiang	Kappa	Lin	Jiang	Kappa	Lin	Jiang	Kappa	Lin	Jiang	Kappa	Lin
CYC2008	Collins	0.6	0.5	0.6	0.5	0.8	0.1	0.1	0.5	0.2	0.1	0.1	0.6	0.5	0.6
	Gavin	0.9	0.4	0.8	0.6	0.5	0.6	0.5	0.6	0.4	0.4	0.3	0.6	0.5	0.7
	Krogan core	0.3	0.4	0.1	0.1	0.1	0.4	0.3	0.5	0.1	0.2	0.3	0.4	0.3	0.6
	Krogan extended	0.5	0.6	0.6	0.4	0.6	0.1	0.4	0.6	0.5	0.4	0.1	0.6	0.4	0.6
	DIP	0.5	-	0.6	0.4	0.5	0.4	0.6	0.5	0.2	0.4	0.6	0.3	0.3	0.6
	BioGRID	0.6	-	0.6	0.6	0.6	0.6	0.6	0.6	0.3	0.4	0.6	0.5	0.1	0.8
SGD	Collins	0.6	0.3	0.6	0.5	0.1	0.1	0.5	0.8	0.3	0.1	0.1	0.6	0.4	0.6
	Gavin	0.9	0.4	0.8	0.5	0.6	0.6	0.8	0.6	0.1	0.4	0.1	0.7	0.4	0.7
	Krogan core	0.3	0.5	0.1	0.4	0.1	0.5	0.3	0.3	0.1	0.3	0.3	0.1	0.3	0.5
	Krogan extended	0.4	0.6	0.4	0.6	0.6	0.1	0.2	0.6	0.5	0.4	0.4	0.4	0.4	0.6
	DIP	0.5	-	0.6	0.4	0.6	0.4	0.6	0.4	0.3	0.6	0.6	0.6	0.3	0.6
	BioGRID	0.6	-	0.8	0.7	0.6	0.6	0.9	0.6	0.6	0.4	0.6	0.5	0.1	0.6

References

[1] B. Adamcsek et al. Cfinder: locating cliques and overlapping modules in biological networks. *Bioinformatics*, 22(8):1021 – 1023, 2006.

[2] Yong-Yeol Ahn, James P Bagrow, and Sune Lehmann. Link communities reveal multiscale complexity in networks. *Nature*, 466(7307):761–764, 2010.

[3] M. Ashburner et al. Gene ontology: tool for the unification of biology. *Nat. Genet.*, 25(1):25–29, 2000.

[4] Gary D Bader and Christopher WV Hogue. An automated method for finding molecular complexes in large protein interaction networks. *BMC Bioinformatics*, 4(1):2, 2003.

[5] Elizabeth I Boyle, Shuai Weng, Jeremy Gollub, Heng Jin, David Botstein, J Michael Cherry, and Gavin Sherlock. Go:: Termfinderłopen source software for accessing gene ontology information and finding significantly enriched gene ontology terms associated with a list of genes. *Bioinformatics*, 20(18):3710–3715, 2004.

[6] S. Brohée and J. Van Helden. Evaluation of clustering algorithms for protein-protein interaction networks. *BMC Bioinformatics*, 7(1):488, 2006.

[7] Deng Cai, Xiaofei He, Jiawei Han, and Thomas S Huang. Graph regularized nonnegative matrix factorization for data representation. *IEEE Transactions on Pattern Analysis and Machine Intelligence*, 33(8):1548–1560, 2011.

[8] Andrew Chatr-aryamontri et al. The biogrid interaction database: 2013 update. *Nucleic Acids Res.*, 41(D1):D816–D823, 2013.

[9] J.M. Cherry, C. Adler, C. Ball, S.A. Chervitz, S.S. Dwight, et al. SGD: Saccharomyces genome database. *Nucleic Acids Res.*, 26(1):73–79, 1998.

[10] S.R. Collins, P. Kemmeren, X.C. Zhao, J.F. Greenblatt, F. Spencer, et al. Toward a comprehensive atlas of the physical interactome of Saccharomyces cerevisiae. *Mol Cell Proteomics*, 6(3):439–450, 2007.

[11] A.J. Enright et al. An efficient algorithm for large-scale detection of protein families. *Nucleic Acids Res.*, 30(7):1575–1584, 2002.

[12] Illés Farkas, Dániel Ábel, Gergely Palla, and Tamás Vicsek. Weighted network modules. *New Journal of Physics*, 9(6):180, 2007.

[13] Brendan J. Frey and Delbert Dueck. Clustering by passing messages between data points. *Science*, 315(5814):972–976, 2007.

[14] A. C. Gavin et al. Proteome survey reveals modularity of the yeast cell machinery. *Nature*, 440(7084):631–636, 2006.

[15] Peng Jiang and Mona Singh. Spici: a fast clustering algorithm for large biological networks. *Bioinformatics*, 26(8):1105 – 1111, 2010.

[16] Alex T. Kalinka and Pavel Tomancak. linkcomm: an r package for the generation, visualization, and analysis of link communities in networks of arbitrary size and type. *Bioinformatics*, 27(14):2011–2012, 2011.

[17] N. J. Krogan et al. Global landscape of protein complexes in the yeast Saccharomyces cerevisiae. *Nature*, 440(7084):637–643, 2006.

[18] H.W. Kuhn and A.W. Tucker. Nonlinear programming. In *Proceedings of the second Berkeley symposium on mathematical statistics and probability*, volume 1, pp. 481–492. California, 1951.

[19] Daniel D. Lee and H. Sebastian Seung. Algorithms for non-negative matrix factorization. In *Advances in neural information processing systems*, volume 13, pp. 556–562, 2001.

[20] Xiaoli Li et al. Computational approaches for detecting protein complexes from protein interaction networks: a survey. *BMC Genomics*, 11(Suppl 1):S3, 2010.

[21] T. Nepusz et al. Detecting overlapping protein complexes in protein-protein interaction networks. *Nat. Methods*, 9(5):471–472, 2012.

[22] Gergely Palla et al. Uncovering the overlapping community structure of complex networks in nature and society. *Nature*, 435(7043):814–818, 2005.

[23] Shuye Pu et al. Up-to-date catalogues of yeast protein complexes. *Nucleic Acids Res.*, 37(3):825–831, 2009.

[24] Kahn Rhrissorrakrai and Kristin C Gunsalus. Mine: module identification in networks. *BMC Bioinformatics*, 12(1):192, 2011.

[25] Lukasz Salwinski et al. The database of interacting proteins: 2004 update. *Nucleic Acids Res.*, 32(suppl 1):D449–D451, 2004.

[26] Venu Satuluri, Srinivasan Parthasarathy, and Duygu Ucar. Markov clustering of protein interaction networks with improved balance and scalability. In *Proceedings of the First ACM International Conference on Bioinformatics and Computational Biology*, pp. 247–256. ACM, 2010.

[27] D Seung. Algorithms for non-negative matrix factorization. *Advances in neural information processing systems*, 13:556–562, 2001.

[28] Yu-Keng Shih and Srinivasan Parthasarathy. Identifying functional modules in interaction networks through overlapping markov clustering. *Bioinformatics*, 28(18):i473–i479, 2012.

[29] Michael E Smoot et al. Cytoscape 2.8: new features for data integration and network visualization. *Bioinformatics*, 27(3):431–432, 2011.

[30] J. Song and M. Singh. How and when should interactome-derived clusters be used to predict functional modules and protein function? *Bioinformatics*, 25(23):3143–3150, 2009.

[31] Yijia Zhang et al. Construction of ontology augmented networks for protein complex prediction. *PLoS ONE*, 8(5):e62077, 2013.
